# Retinal metabolic events in preconditioning light stress as revealed by wide-spectrum targeted metabolomics

**DOI:** 10.1007/s11306-016-1156-9

**Published:** 2017-01-20

**Authors:** Juan Manuel Chao de la Barca, Nuan-Ting Huang, Haihan Jiao, Lydie Tessier, Cédric Gadras, Gilles Simard, Riccardo Natoli, Guillaume Tcherkez, Pascal Reynier, Krisztina Valter

**Affiliations:** 1 PREMMi/Pôle de Recherche et d’Enseignement en Médecine Mitochondriale, Institut MITOVASC, CNRS 6214, INSERM U1083, Université d’Angers, 49933 Angers, France; 2 Département de Biochimie et Génétique, Centre Hospitalier Universitaire, 4 rue Larrey, 49933 Angers cedex 9, France; 3 INSERM U1063, Université d’Angers, 49933 Angers, France; 4 Eccles Institute of Neuroscience, John Curtin School of Medical research, Australian National University, Canberra, ACT 2601 Australia; 5 Medical School, Australian National University, Canberra, ACT 2601 Australia; 6 Research School of Biology, College of Medicine, Biology and Environment, Australian National University, Canberra, ACT 2601 Australia

**Keywords:** Metabolomics, Retina, Preconditioning, Immunohistochemistry

## Abstract

**Introduction:**

Light is the primary stimulus for vision, but may also cause damage to the retina. Pre-exposing the retina to sub-lethal amount of light (or preconditioning) improves chances for retinal cells to survive acute damaging light stress.

**Objectives:**

This study aims at exploring the changes in retinal metabolome after mild light stress and identifying mechanisms that may be involved in preconditioning.

**Methods:**

Retinas from 12 rats exposed to mild light stress (1000 lux × for 12 h) and 12 controls were collected one and seven days after light stress (LS). One retina was used for targeted metabolomics analysis using the Biocrates p180 kit while the fellow retina was used for histological and immunohistochemistry analysis.

**Results:**

Immunohistochemistry confirmed that in this experiment, a mild LS with retinal immune response and minimal photoreceptor loss occurred. Compared to controls, LS induced an increased concentration in phosphatidylcholines. The concentration in some amino acids and biogenic amines, particularly those related to the nitric oxide pathway (like asymmetric dimethylarginine (ADMA), arginine and citrulline) also increased 1 day after LS. 7 days after LS, the concentration in two sphingomyelins and phenylethylamine was found to be higher. We further found that in controls, retina metabolome was different between males and females: male retinas had an increased concentration in tyrosine, acetyl-ornithine, phosphatidylcholines and (acyl)-carnitines.

**Conclusions:**

Besides retinal sexual metabolic dimorphism, this study shows that preconditioning is mostly associated with re-organisation of lipid metabolism and changes in amino acid composition, likely reflecting the involvement of arginine-dependent NO signalling.

**Electronic supplementary material:**

The online version of this article (doi:10.1007/s11306-016-1156-9) contains supplementary material, which is available to authorized users.

## Introduction

Light radiation in wavelengths of the visible spectrum is the primary stimulus for vision and is thus essential for retinal function. However, light can also induce retinal damage (Noell et al. 1966; Rutar et al. 2010). Although there is no clear direct evidence that light causes irreparable retinal degradation over a human being’s lifetime, it is widely believed that oxidative stress and activation of the immune system caused by long-term exposure to moderate light, in addition to ageing (i.e. metabolic and circulatory changes, and immune-senescence) can lead to progressive tissue damage in the retina (Natoli et al. 2016; Organisciak et al. 1998; Penn and Anderson 1987; Rutar et al. 2011, 2012; Youssef et al. 2011). Retina’s prior light-experience (so-called ‘light history’) has been shown to be essential for determining its sensitivity to subsequent acute light damage, suggesting that retinal cells may acclimate to their environment thereby maximizing their ability to survive on the long-term (Organisciak et al. 1998; Penn and Anderson 1991). Organisciak and coworkers have demonstrated that the retina is more susceptible to light damage if exposed to intense light during nighttime and conversely, more resilient to light damage when intense light exposure occurs during daytime (Organisciak et al. 2000). In addition, pre-exposure to moderate light stress (referred to as ‘preconditioning’) increases retina’s resilience to subsequent intense light imposed during either nighttime or daytime.

During preconditioning, the retina has been reported to undergo biochemical changes to prevent or mitigate the formation of reactive oxygen species and/or to increase its ability to alleviate their effects (Penn et al. 1987; Penn and Anderson 1987). Considering the intense membrane dynamics in photoreceptors and ancillary cells types (e.g. retinal pigment epithelium), such changes probably include membrane lipids reorganization and protein recycling. However, a comprehensive examination of metabolic changes associated with preconditioning is still needed, to have a deeper insight into the process of retinal adaptation.

Here, we took advantage of wide-spectrum targeted metabolomics techniques to provide an overview of retinal metabolic composition and investigated metabolic events that occur during preconditioning. Metabolomics in retinal biochemistry has been recognized to be instrumental in elucidating the mechanisms and predict outcome severity of eye diseases, or in identifying potential therapeutic targets (Tan et al. 2016). In practice, the detection of early changes in the metabolic composition of light-stressed retinal tissue can help describing cellular responses and thus identifying key features for retinal damage monitoring. In the present paper, we subjected albino rats (both males and females) to moderately light stress (1000 lux × for 12 h) during daytime, so as to reproduce a typical preconditioning situation. We then used liquid chromatography/mass spectrometry (LC-MS) to follow retinal metabolomics patterns, and monitored cellular changes by immunohistochemistry.

## Materials and methods

### Experimental protocol

**Fig. 1:**
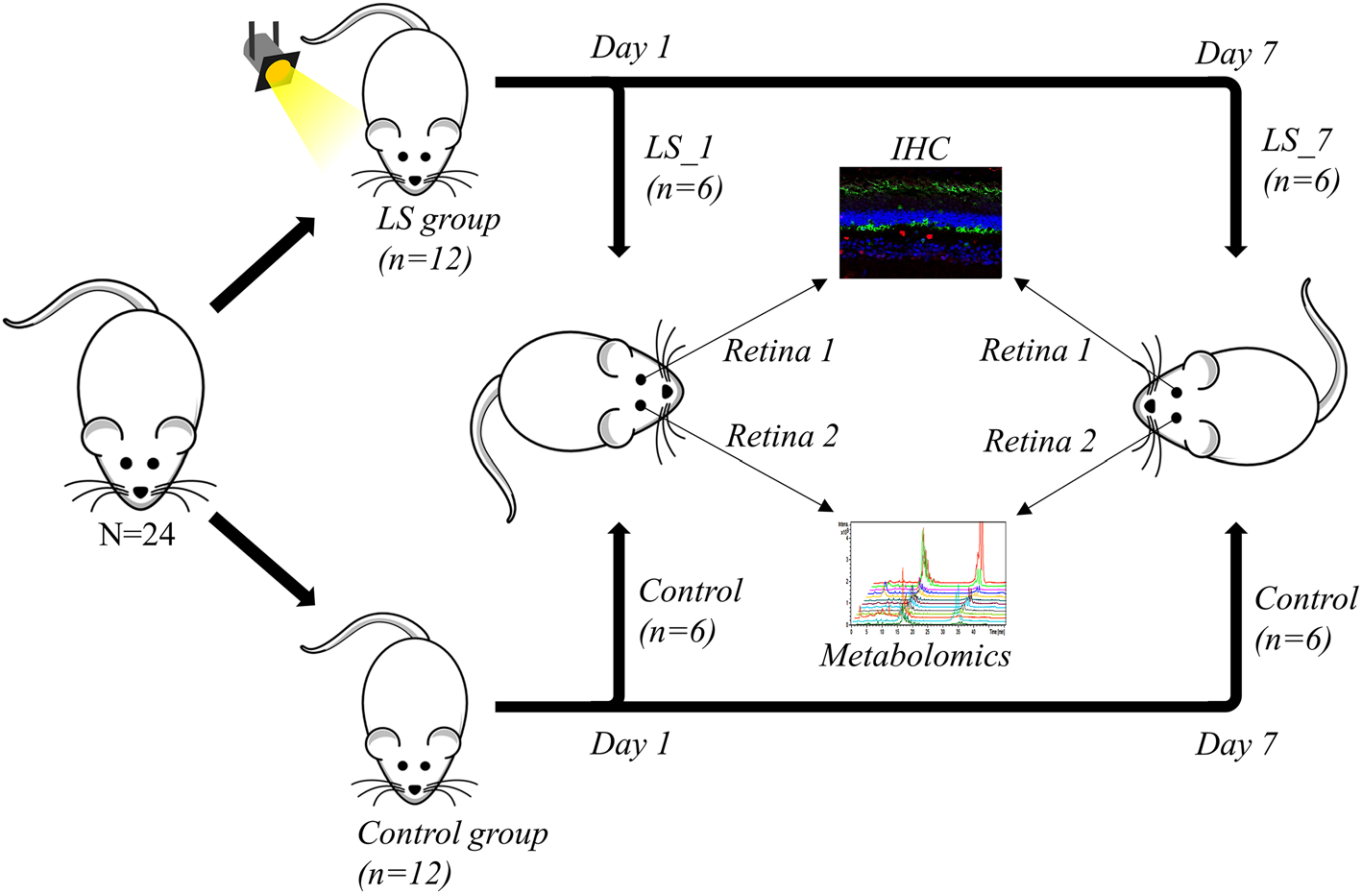
Experimental design: 6 of the 12 rats undergoing light stress (LS group) were culled 1 day after LS (LS_1 group) while the other 6 were culled 1 week after LS (LS_7 group). Six non-exposed rats were sacrificed at the same sampling time (control group). After culling, one eye was randomly chosen for immunohistochemistry (IHC) analysis whilst the retina of the fellow eye was used for the metabolomics analysis

All experiments conducted were in accordance with the ARVO Statement for Use of Animals in Ophthalmic and Vision Research; the study including the number of rats used was approved by the Animal Experimentation Ethics Committee (AEEC) of the Australian National University (R.BSB.05.10). Thirteen female (F) and 11 male (M) Sprague–Dawley albino rats (*n* = 24) aged from 112 to 129 days, reared in dim cyclic light (12 h/12 h) were used. Twelve animals (LS group, F/M = 7/5) were exposed to 1000 lux for 12 h (Fig. 1). Twenty-four hours after the commencement of light exposure, 6 animals (F/M = 4/2) were returned to normal dim cyclic light environment for 7 days, while the other 6 animals (F/M = 3/3) and 6 non-exposed controls (F/M = 3/3) were culled by CO_2_ and collection started (LS_1 and C_1 groups, respectively). The retina from one randomly chosen eye was collected and put in a 0.5 mL cooled Precellys® tube pre-filled with ceramic beads and stored at −80 °C until prepared for metabolomics analyses. The fellow eye was enucleated for immunohistochemical (IHC) analysis. The remainder 6 light-exposed (F/M = 4/2) animals and 6 controls (F/M = 3/3) animals were culled by CO_2_ 7 days after light stress and eyes were processed following the same protocol as in the first group (LS_7 and C_7 groups, respectively).

### Histology and immunohistochemical (IHC) analysis

Retinal morphology was visualized using hematoxylin and eosin and fluorescein DNA staining, cell damage and apoptosis was detected by TUNEL, cell stress and immune response were monitored by IHC using antibodies anti-GFAP (glial fibrillary acidic protein) and anti-IBA-1 (ionized calcium binding adaptor molecule 1), respectively. Along with immunohistochemical labeling, DNA was stained with bisbenzimide (BBZ). The protocol used for tissue collection, histological and IHC analysis is described in Supplementary Methods S1.

### Metabolomics analysis

#### Sample processing

Samples stored at −80 °C were thawed on ice and 40 µL of MilliQ water were added. Retina homogenization was achieved using a Precellys®24-Cryolys homogenizer (Bertin, France) by two cycles of grinding (40 s at 6500 rpm, followed by 30 s at 6000 rpm) at 4 °C. The resulting homogenate was centrifuged at 12,000 g for 5 min at 4 °C and 5 µL of the supernatant were transferred in an Eppendorf tube containing 25 µL of distilled water for protein quantitation. 140 µL of methanol were added to the Precellys tube and another grinding cycle (40 s at 6500 rpm) was done. After centrifugation at 16,000*g* for 5 min, 140 µL of the supernatant were transferred to a second Eppendorf tube and spin-dried for 24 h. Extracts were kept at −80 °C until metabolomics analyses.

#### Mass spectrometry assay

Targeted quantitative metabolomics analyses were carried out using the Biocrates Absolute IDQ p180 kit (Biocrates Life sciences AG, Innsbruck, Austria). This kit, combined with a QTRAP 5500 (SCIEX, Villebon sur Yvette, France) mass spectrometer, enables quantification of up to 188 different endogenous molecules including hydrophilic and less polar metabolites (the full list is available in Supplementary Table S2). Flow injection analysis (FIA-MS/MS) was used for quantifying carnitine, acylcarnitines, lipids and sugar, whilst liquid chromatography (LC) was employed to separate amino acids and biogenic amines before MS quantitation. All reagents used in this analysis were of LC-MS grade and purchased from VWR (Fontenay-sous-Bois, France) and Merck (Molsheim, France). Sample preparation was performed according to the Kit User Manual. Briefly, for each sample 30 µL of methanol were added, extracts were vortexed thoroughly for 5 min, then 10 µL were mixed with isotope-labeled internal standards and samples were loaded onto the 96-well plate. Metabolites were re-suspended in ammonium acetate after filter spots have been dried under nitrogen flow and derivatized with phenylisothiocyanate (only for amino acids and biogenic amines quantitation). Extracts were diluted with MS running solvent (MilliQ water for HPLC assay or a methanol solution for FIA assay) prior to FIA and LC-MS/MS analyses. Quality controls (QC) at three concentrations (referred to as low, medium and high) were included in the kit and analyzed along with samples. Values of the coefficient of variation (CV = standard deviation/mean × 100, in %) associated with QC samples were used to validate quantitation in samples (CV threshold of 30 %). The software Analyst (SCIEX) was used for MS data collection and the software MetIDQ (Biocrates) was used to monitor the entire assay workflow.

### Statistical analysis

Before performing statistical analysis, raw data were examined in order to eliminate metabolites that appeared not to be accurately measured, i.e., metabolites with a concentration that was below the lower (LLOQ) or above the upper limit of quantitation (ULOQ). When more than 20 % of concentration values were below the lower limit of quantitation or above the upper limit of quantitation, the metabolite of interest was not considered for statistical analyses (the list of metabolites that were excluded thereby is tabulated in Supplementary Table S6). In order to account for differences in metabolite concentration due merely to differences in the mass of retina extracted (in practice, it proved difficult to weight accurately tiny tissue samples like retina), we used soluble protein concentration as a surrogate for retina weight. That is, metabolomics data were normalized to protein content prior to statistical processing. Original, non-normalized data and protein contents are available in Supplementary Table S3. Univariate analysis was made using the non-parametric Wilcoxon rank sum test (here simply referred to as “Wilcoxon test”) for comparisons involving two independent samples, and the non-parametric Kruskal–Wallis test for comparisons between more than two independent samples. A *p* value less than 0.05 was considered to be statistically significant unless otherwise mentioned. When applicable (Supplementary Tables S4 and S5), *p* value threshold correction for false discovery rate (FDR) was made as in (Tan and Xu 2014). Univariate analyses were conducted using R software, version 3.2.1 (http://www.R-project.org).

Multivariate analyses were performed using unit variance-scaled data. Principal components analysis (PCA) was used to detect sample groups and outliers. An orthogonal partial least squares discriminant analysis (OPLS-DA), which is a supervised pattern recognition method, was then performed to maximize variation between groups and to determine variables contributing to this variation. The quality of models was validated using two parameters: R² (goodness of fit) and cumulated Q² (Q²_cum_, goodness of prediction). A threshold of 0.5 is widely accepted in model classification to identify good (Q²_cum_ ≥0.5) or poor (Q²_cum_ <0.5) predictive capabilities. The risk of over-fitting and robustness was assessed by the intercept of the permutation plot and the cross validated ANOVA (CV-ANOVA), respectively. This intercept reflects the predictive capabilities (*permQ* ^*2*^) of a model having the same number of components (or latent variables) and the same X matrix (metabolites) but a randomly permuted Y vector (scrambled Y) of response variables (that is, at the intercept, there is no correlation between the original Y vector and the permuted Y vector). Non over-fitted models have negative *permQ* ^*2*^ value (Umetrics 2013). In the CV-ANOVA test, the CV predicted residuals using the OPLS model are compared, using an ANOVA test, to the residuals obtained as a variation around the Y-average. When the null hypothesis of equality of the two residual (*F* test) cannot be rejected (*p* value > 0.05 here), the equality of the OPLS and a random model cannot be rejected (Eriksson et al. 2008). In the model with the best predictive capabilities, key variables were highlighted based on the variable importance for the projection (VIP) and loading values scaled as correlation coefficients (p_corr_). VIP values summarize the importance of each variable for the OPLS-DA model, while loadings are indicators of the relationship between Y (sex, light damage) and X variables (matrix of measured metabolites). Variables having a VIP value higher than 1 are considered to be important for group discrimination in predictive models (Eriksson et al. 2006). Plotting VIP vs. p_corr_ values (“volcano” plot) enabled highlighting important variables in PLS models. Multivariate data analysis was conducted using SIMCA-P v. 14.0 (Umetrics, Umeå, Sweden).

## Results

### Retinal histology and IHC

**Fig. 2:**
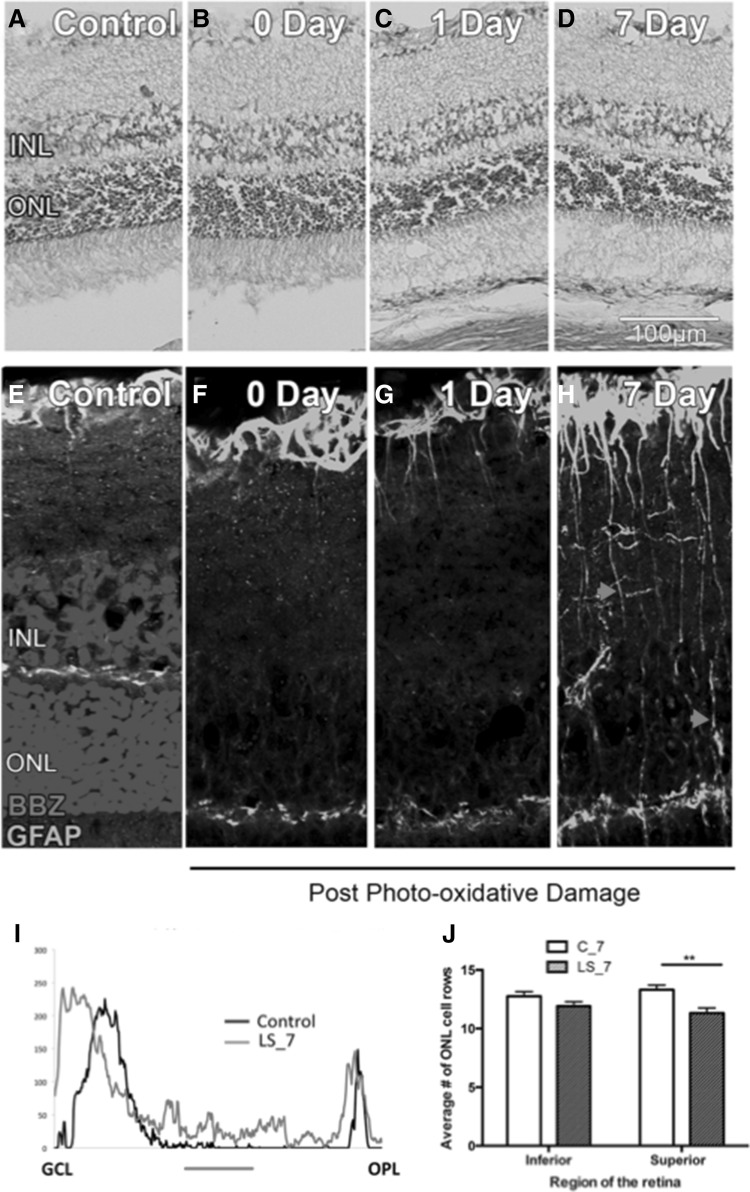
Histology (**a**–**d**) and glial fibrillary acidic protein (GFAP) immunohistochemistry (**e**–**h**) of the retina of controls after 1 day (C_1, **a, b** and **e, f**), light stressed retinas (LS), 1 day (**c** and **g**) or 7 days (**d** and **h**) after light exposure. **e**–**h** GFAP (green fluorescence) was present in the astrocyte layer in the control retina (**e** and **f**). In **e**, cell nuclei were stained with BBZ. One day after LS, some Muller cell processes were shown to express the protein but this expression was localized close to the astrocyte layer (**g**). By 7 days post-LS GFAP labeling was evident in a large number of Muller cell processes that are spanning across the inner retina (**h**, *red arrow heads*). Panel **i** shows increased expression of GFAP in the inner plexiform layer (*red bar*). There was a significant reduction in ONL thickness in the superior retina by 7 days post-LS (**j**)

**Fig. 3:**
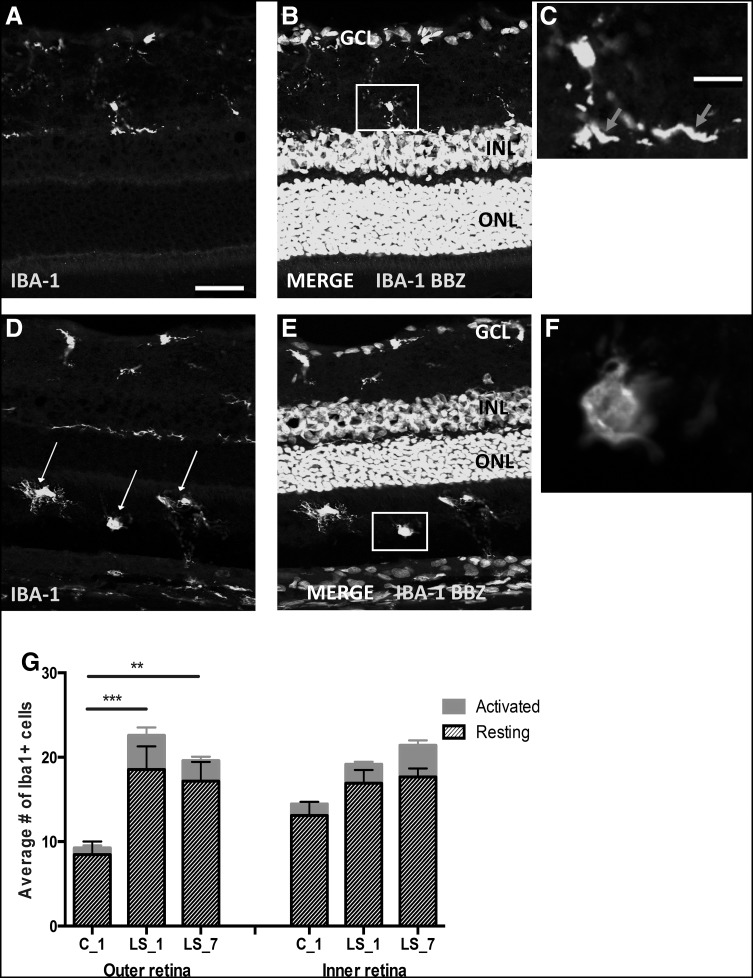
Immunohistochemical cross-section of retina in controls (**a**–**c**) or 7 days after light stress (d–f), showing fluorescence of anti-IBA-1 (*green*) antibody and BBZ (*turquoise*) staining. In the control retina, an IBA-1^+^ cell in the inner retina (the part of the neuroretina including the inner nuclear, inner plexiform and ganglion cell layers) has a ramified morphology indicative of resting microglia (magnified in *inset* in **c**, *red arrows*). Note the absence of IBA-1^+^ cells in the ONL (outer retina). In LS_7, there is an increased number of IBA-1^+^ cells and many of those are apparent in the outer retina (*white arrows* in **d**). In **e**, an example of an IBA-1^+^ cell in the outer retina with an amoeboid morphology of activated microglia (magnified in *inset* in **f**). *Scale bar* 100 µm (**a, b, d** and **e**) or 10 µm (**c, f**). The *graph* in **g** confirmed an increased number of both, resting and activated microglia in the outer retina after LS. This increase was significant for both overall (activating + resting) and resting microglia, in the outer retina only

Histological analysis of the retinas showed no significant pathological changes after LS (Fig. 2a–d). IHC analysis of GFAP expression showed a redistribution of protein expression. In control retinas, GFAP was detected in astrocytes while in light-stressed retinas, GFAP was also detected in Müller cells (Fig. 2e–i). This change in GFAP labeling is a well-accepted sign of tissue stress. There was a significant increase in the numbers of cells prone to apoptosis (i.e. TUNEL positive) 1 day after light exposure (Fig. 2j) that was associated with a small reduction in photoreceptor cell numbers in superior retina 7 days after light exposure (Fig. 2k). TUNEL counts are not shown for the LS_7 group, since there was no TUNEL+ profile found in any region of the retina. No TUNEL+ profiles were detected in the inner retinal layers (data not shown). One day after LS, IHC with anti-IBA-1 antibody showed that there was an increase in the number of microglia and activated microglia/macrophages appeared in the outer retina, where they are normally absent (Fig. 3). Neither histological nor IHC parameters were different between controls at 1 and 7 days. Also, ONL thickness did not show any change after 1 day, which agrees with previous findings in light-stressed rat retina (Liu et al. 1998). Taken as a whole, these results show a mild light stress with minimal loss of photoreceptors, but with a moderate activation of the microglia-Müller cells response, typical of preconditioning.

It is worth noting that here, we report on focal retinal damage, typical of the present photo-oxidative model. Mild histological changes were observed 300 µm supero-temporal to the optic disc (*area centralis* of the rat retina), while there were no observable changes in the peripheral retina. Despite the relatively moderate focal damage, we were able to detect measurable metabolic changes using the whole retina in our analyses (see below).

### Overview of metabolomics analyses

After plate validation based on QC samples, 165 (87.8 %) metabolites were retained for the statistical analyses. Raw data with the full list of metabolites are available in **Supplementary Table S3**. The mean soluble protein concentration was not significantly different (1) between controls, LS_1 and LS_7 groups (1.51, 1.01 and 1.16 g L^− 1^, respectively, Kruskal–Wallis test, *p* = 0.12), or (2) between males and females when considering all rats (1.43 vs. 1.21 g L^− 1^, Wilcoxon test, *p* = 0.28) or only the control group (1.48 vs. 1.58 g L^− 1^, *p* = 0.94).

### Metabolomics sexual dimorphism between female and male retinas

**Fig. 4:**
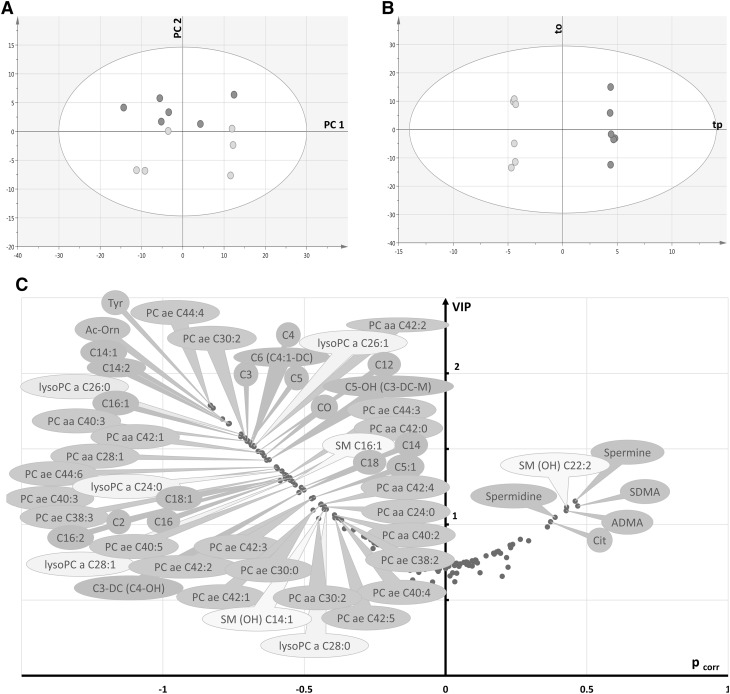
Multivariate analysis of control retinas: **a** first principal plan of the PCA (axis 1 vs. axis 2) showing no clear outlier and a separation between males (*green circles*) and females (*pink circles*), **b** OPLS-DA scatter plot showing a clear discrimination between the two groups along the predictive component *tp*. **c** Volcano plot of metabolites that best discriminate groups. They are essentially belonging to glycerophospholipids (*dark* and *light orange* bubbles) and acylcarnitines (*brown* bubbles), both relatively increased in male retinas, along with some sphingomyelins (*yellow* bubbles), amino-acids and biogenic amines (*green* bubbles). *Legend*: (in what follows, X indicates the length of the acyl chain and Y the degree of unsaturation) *CX:Y* acyl-l-carnitines, *PC aa CX:Y* phosphatidylcholine diacyl, *PC ae CX:Y* phosphatidylcholine acyl-alkyl, *lysoPC a CX:Y* lysophosphatidylcholine acyl, *SM CX:Y* sphingomyelin, *SM(OH) CX:Y* hydroxysphingomyelin, *C0* carnitine, *C2* acetyl-l-carnitine, *C3* propionyl-l-carnitine, *C3-DC (C4–OH)* malonyl-l-carnitine (hydroxybutyryl-l-carnitine), *C4* butyryl-l-carnitine, *C5* valeryl-l-carnitine, *C5:1* tiglyl-l-carnitine, *C5–OH (C3-DC-M)* hydroxyvaleryl-l-carnitine (methylmalonyl-l-carnitine), *C6 (C4:1-DC)* hexanoyl-l-carnitine, *C12* dodecanoyl-l-carnitine, *C14* tetradecanoyl-l-carnitine, *C14:1* tetradecenoyl-l-carnitine, *C14:2* tetradecadienyl-l-carnitine, *C16* hexadecanoyl-l-carnitine, *C16:1* hexadecenoyl-l-carnitine, *C16:2* hexadecadienyl-l-carnitine, *C18* octadecanoyl-l-carnitine, *C18:1* octadecenoyl-l-carnitine, *Ac-orn* acetylornithine, *ADMA* asymmetric dimethylarginine, *Cit* citrulline, *SDMA* symmetric dimethylarginine, *Tyr* tyrosine

PCA of control retinas showed no strong outlier and a rough separation between male and female rat retinas in the first principal plane (Fig. 4a). The OPLS-DA model fitted well the data (R^2^X = 0.91, R^2^Y = 0.99) and had excellent predictive capabilities (Q²_cum_ = 0.95) with a low risk of over-fitting (*permQ* ^*2*^ = − 0.55, CV-ANOVA = 0.015). A clear discrimination between the two groups along the predictive component (*tp*) was apparent in the scatter plot of the OPLS-DA model (Fig. 4b). Best discriminant metabolites included those related with lipid metabolism, as well as some amino acids and biogenic amines (Fig. 4c). Metabolic ratios were computed in both groups (Supplementary Table S4) thereby indicating potential enzymatic activities. Phenylalanine hydroxylase activity was significantly higher in male retinas according to the tyrosine-to-phenylalanine ratio (1.99 vs. 1.46, *p* = 0.025). Nitric oxide (NO) synthase activity (citrulline-to-arginine ratio) tended to be higher in female retinas (*p* = 0.055) but the increased ADMA content in female retinas (volcano plot, Fig. 4c) might have counteracted this potential increase in enzyme activity (*p* = 0.078). In lipid metabolism, an enhanced activity of β- and ω-oxidation of fatty acids was found in female compared to male control retinas (*p* = 0.015 and *p* = 0.025, respectively).

### Effect of light stress on retinal metabolome

**Fig. 5:**
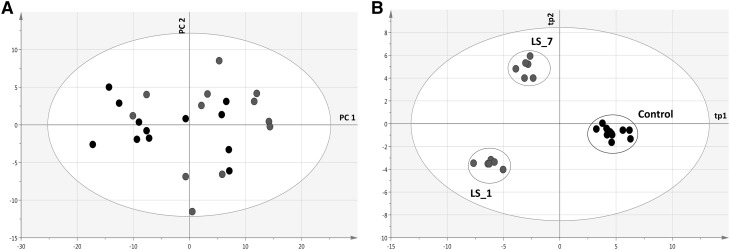
Multivariate analysis of control and LS retinas: **a** first principal plane (axis 1 vs. axis 2) of the PCA scatter plot showing no outlier and no separation between controls (*black circles*) and retinas 1 (LS_1) or 7 (LS_7) d after light treatment (*blue* and *red circles*, respectively), **b** OPLS-DA scatter plot showing a clear discrimination between groups according to light treatment along the predictive components *tp*1 and *tp*2

To carry out multivariate statistical analyses, light stress was encoded as a categorical response vector Y with three possible values: controls, 1 day (LS_1) and 7 days after light stress (LS_7). In fact, we gathered controls at 1 and 7 days within the same group since there were no differences in histology and IHC between them and multivariate analysis failed to separate them (data not shown). The PCA showed no outlier and no sample grouping in the first principal plan (Fig. 5a). The OPLS-DA yielded a model that fitted well the data (R^2^X = 0.92, R^2^Y = 0.98) and had good predictive capabilities (Q²_cum_ = 0.75) with a low risk of over-fitting (*permQ* ^*2*^ = − 0.98, CV-ANOVA = 0.026). A clear discrimination between samples was achieved along the first (*tp*1) and the second (*tp*2) predictive latent variables of the model (Fig. 5b). The first predictive component (*tp*1) mostly discriminated between control and light-treated retinas (LS_1 and LS_7) while the second predictive component (*tp*2) discriminated between LS_1 and LS_7 retinas. Therefore, top metabolites (i.e., VIP ≥ 1) could be sorted according to their loading (p_corr_) along *tp*1 and/or *tp*2 component (p_corr_1 and p_corr_2, respectively).

**Fig. 6:**
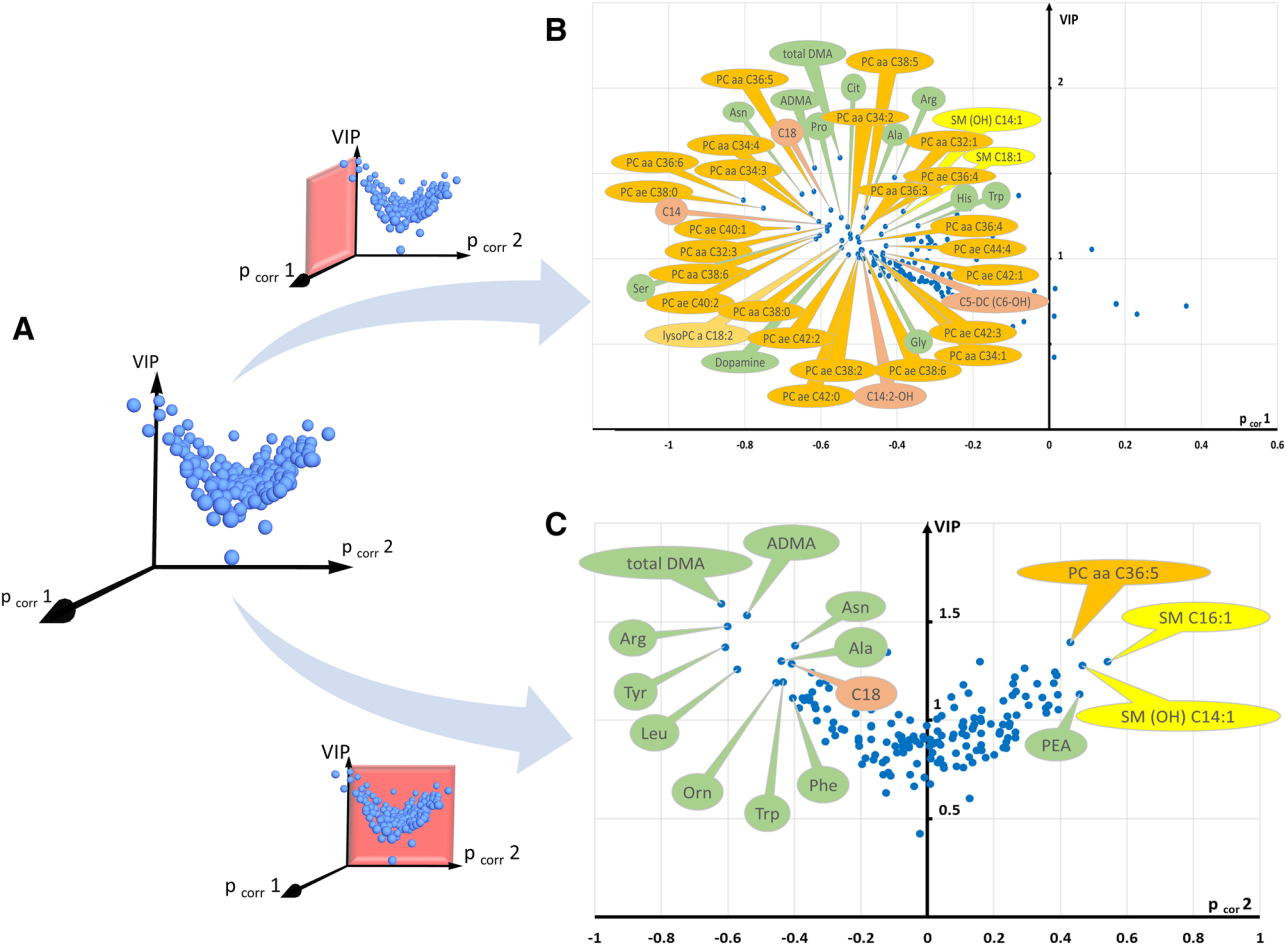
3D- and 2D-Volcano plots of metabolites that best discriminate between controls, LS_1 and LS_7 groups: **a** Metabolites (*blue spheres*) form a volcano-shaped cloud in a 3D system of coordinates formed by p_corr_1, p_corr_ 2 and VIP axes. Top discriminant metabolites along *tp*1 (light stressed vs. control retinas) and *tp*2 (retinas 1 vs. 7 days after LS) axes are displayed by projecting the cloud along the VIP *vs*. p_corr_1 plane (**b**) or along the VIP vs. p_corr_ 2 plane (**c**), respectively (highlighted in *pink*). In **b**, all important metabolites have negative p_corr_ 1 values indicating their relatively increased concentration after LS. These metabolites are mainly phosphatidylcholines (*dark orange* bubbles), amino acids and biogenic amines (*green* bubbles) along with 2 sphingomyelins (*yellow* bubbles) and 4 acylcarnitines (*brown* bubbles). In **c**, metabolites discriminating between LS_1 (negative p_corr_ 2 values) and LS_7 (positive p_corr_ 2 values) groups are mainly amino acids, asymmetric and total dimethylarginine (ADMA and total DMA, respectively), phenylethylamine (PEA), 2 sphingomyelins, 1 phosphatidylcholine and octadecanoyl-l-carnitine (C18). *Legend*: (in what follows, X indicates the length of the acyl chain and Y the degree of unsaturation) *CX:Y* acyl-l-carnitines, *PC aa CX:Y* phosphatidylcholine diacyl, *PC ae CX:Y* phosphatidylcholine acyl-alkyl, *lysoPC a CX:Y* lysophosphatidylcholine acyl, *SM CX:Y* sphingomyelin, *SM(OH) CX:Y* hydroxysphingomyelin, *C5-DC (C6–OH)* glutaryl-l-carnitine, *C14* tetradecanoyl-l-carnitine, *C14:2–OH* hydroxytetradecadienyl-l-carnitine, *C18* octadecanoyl-l-carnitine, *ADMA* asymmetric dimethylarginine, *Ala* alanine, *Arg* arginine, *Asn* asparagine, *Cit* citrulline, *Gly* glycine, *His* histidine, *Orn* ornithine, *PEA* phenylethylamine, *Pro* proline, *Ser* serine, *total DMA* total dimethylarginine, *Trp* tryptophan

As compared to controls, light-treated retinas showed an increase in 24 phosphatidylcholines, lysophosphatidylcholine C18:2, 4 acyl-carnitines (octadecanoyl-, tetradecanoyl-, hydroxytetradecadienyl-, and glutaryl-l-carnitine), 9 amino acids (asparagine, proline, arginine, alanine, serine, histidine, glycine, tryptophan and citrulline) and total and asymmetric dimethylarginine (total DMA and ADMA, respectively) (Fig. 6b). Metabolites considered to be important to discriminate between LS_1 and LS_7 groups were those having p_corr_2 values ≥ 0.4 or ≤ -0.4 and VIP values ≥ 1 (Fig. 6c). Two mono-unsaturated sphingomyelins (SM C16:1 and SM(OH) C14:1) appeared to be increased in the LS_7 group as compared to the LS_1 group along with one phosphatidylcholine (PC aa 36:5) and phenylethylamine (PEA). Eight amino acids (arginine, alanine, asparagine, leucine, ornithine and aromatics tyrosine, tryptophan and phenylalanine) as well as total DMA and ADMA, were relatively increased in the LS_1 group.

Metabolic ratios were computed in the three sample groups (Supplementary Table S5). A two-fold increase in the citrulline-to-arginine ratio (activity of NO synthase) was observed in LS_7 retinas as compared to LS_1 (*p* = 0.025). The LS_7 group also had an increased saturated-to-monounsaturated PC desaturase activity (*p* = 0.011) combined with decreased saturated-to-monounsaturated and saturated-to-polyunsaturated SM desaturase activities (*p* = 0.029 and *p* = 0.048, respectively). Monounsaturated-to-polyunsaturated lyso-PC desaturase activity was also significantly higher in both LS_1 and LS_7 groups compared to controls (*p* = 0.012).

## Discussion

Pre-exposure to moderate light (preconditioning) before intense light exposure is believed to be protective for retinal photoreceptors. Although some mechanisms have been suggested to explain this protective effect, such as the induction of neuro-protective factors (Liu et al. 1998) or an increase in antioxidant levels (Penn et al. 1987), the metabolic basis of preconditioning has not been explored yet. Here, we have analyzed the metabolomics pattern of rat retina subjected to preconditioning at different time points (1 day, 7 days). In addition to clear preconditioning-induced metabolic effects, we found unexpected differences between male and female retina metabolite contents.

### Sexual dimorphism of rat retinal metabolome

Differences between male and female retina metabolome in controls were found in glycerophospholipids, biogenic amines and amino acids. The most striking difference was associated with lipid metabolism. In fact, male retinas showed a relatively increased carnitine, acylcarnitines and phospholipids concentration, with metabolite ratios involving the carnitine family suggesting an increased fatty acid (FA) oxidation in female retinas (Fig. 4; Supplementary Table S4). FA oxidation requires transport into the mitochondrial matrix, which in turn involves conjugation to carnitine (via carnitine palmitoyl-transferase activity). Free FA originates from the action of the retinal pigment epithelium (RPE) which phagocytes shed tips of photoreceptors, eventually leading to phagolysosomes and, after digestion, to FAs liberation. Thus, the lower (acyl)-carnitine pool in females could have come from higher FA oxidation capacity, larger mitochondriome or up-regulation of FA oxidation by, e.g., hormonal signaling in females. The credibility of such hypotheses is assessed in the Supplementary Text.

### Effect of light treatment on rat retina metabolome

**Fig. 7:**
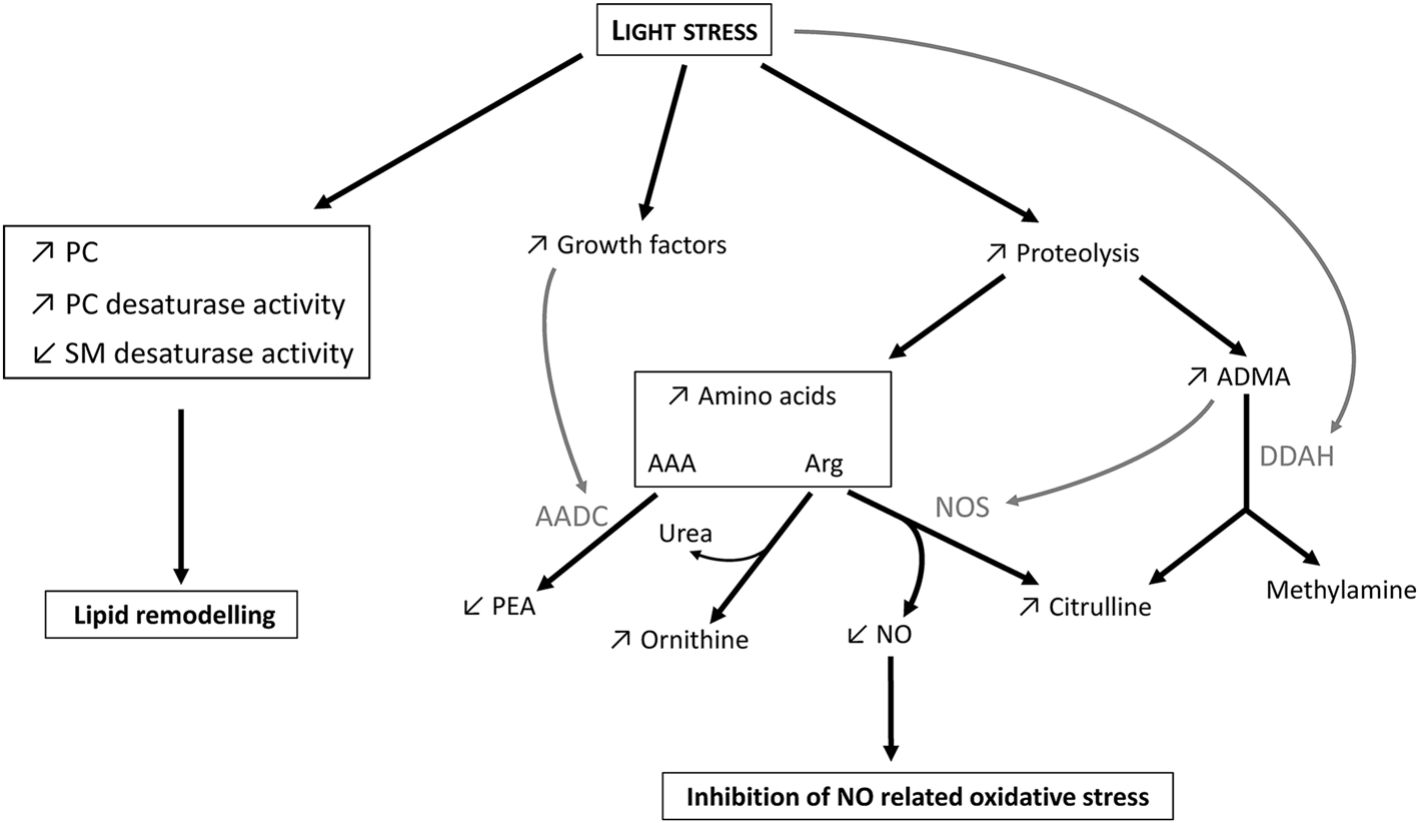
Graphical summary of major metabolic changes observed 1 day after light stress. *AAA* aromatic amino acids, *AADC* aromatic amino acid decarboxylase, *ADMA* asymmetric dimethyl arginine, *DDAH* dimethyl arginine dimethylaminohydrolase, *NOS* NO-synthases, PC phosphaditylcholines, *PEA* phenylethylamine, *SM* shingomyelins. In this figure, the term “growth factors” refers to bFGF, CNTF and LIF. *Arrows* represent observed changes upon light stress (increase, ↗ or decrease, ↘). *Grey arrows* represent inhibition. See the text for further details

Marked changes in rat retinal metabolome were observed after LS (Figs. 5b, 6). They are summarized in Fig. 7. Some of these changes, like an elevated amino acid pool, were more intense 1 day after LS while others, like the increase in a number of phosphatidylcholines, were observed from 1 to 7 days after LS. The intensity of LS applied in our study was sufficient to stimulate Müller cells but caused only minimal photoreceptor loss as assessed by IHC analysis (Fig. 2). Under moderate LS, it is believed that two processes occur, within different time scales (Organisciak and Vaughan 2010). The first process consists in photoreceptor cell damage resulting in enhanced apoptosis and autophagy with the activation of proteolytic enzymes like caspases, cathepsin D and calpains just a few hours after LS (Kunchithapautham and Rohrer 2007). The second process peaks a few days after mild LS and involves photoreceptors repair. It is triggered by neuroprotective factors, like basic fibroblast growth factor (bFGF), ciliary neurotrophic factor (CNTF), glial cell line-derived neurotrophic factor (GDNF) and leukemia inhibitory factor (LIF), coming from the interaction between activated microglia and Müller cells (Chollangi et al. 2009; Harada et al. 2002; Ueki et al. 2008). During this second phase also, the retina becomes more resistant to light-induced damage, that is, develops preconditioning (Li et al. 2001; Liu et al. 1998, see also the Introduction). Preconditioning has been shown to be associated with protein degradation: opsin and rhodopsin autophagic degradation has been shown to alleviate light-induced stress and protect photoreceptors from further damage during light re-exposition (Remé et al. 1999). Here, the increased concentration in 12 amino acids found 1 day after LS (Fig. 6b, c) is likely the consequence of augmented proteolysis such as, typically, proteasomal degradation of some photoreceptor proteins.

Our analyses also show a significant change in metabolites associated with the nitric oxide (NO) pathway. It is well established that NO can be elevated under pathological conditions such as autoimmune uveitis (Hsu et al. 2015), glaucoma, diabetic retinopathy and ischemic retinopathy (reviewed in Toda and Nakanishi-Toda 2007) and retinopathy in prematurity (Rey-Funes et al. 2016). The involvement of NO metabolism upon LS is consistent with its critical role in retinal signaling (Vielma et al. 2012). In mammals, NO synthases (NOS; EC 1.14.13.39) are a family of 3 isoenzymes [neuronal (nNOS), endothelial (eNOS) and inducible (iNOS)] that catalyze the formation of citrulline and NO from arginine (Knowles and Moncada 1994). The 3 isoforms of NOS have been identified in the retina where NO is generated upon dark-to-light transition in photoreceptors and involved in photoreceptor phagocytosis by RPE and Müller cells and the regulation of retinal blood circulation (Vielma et al. 2012). In spite of these beneficial effects in retinal physiology, high NO levels produced by iNOS generate oxidative stress and has been shown to be involved in retinal ischemic injury (Goldstein et al. 1996). We have also previously shown the increase in iNOS in photo-oxidative damage (Albarracin and Valter 2012). Elevated NO content can cause S-nitrosation and peroxynitrate formation leading to further cellular injury and apoptosis (Roth 1997). Donovan et al. also found that inhibition of nNOS prevented light-induced retinal degeneration in mice and human (Donovan et al. 2001).

Asymmetric dimethylarginine (ADMA) is a potent inhibitor of NO synthesis by competing with arginine for the active site of NOS (Vallance et al. 1992). ADMA residue comes from dimethylation of arginine residues in nuclear and cytosolic proteins catalyzed by enzymes of the protein arginine N-methyltransferase (PRMT; EC 2.1.1.125) family (Bedford 2007). ADMA is released during the degradation of proteins containing ADMA residues and is then hydrolyzed to citrulline and dimethylamine by dimethylarginine dimethylaminohydrolases 1 and 2 (DDAH 1 and 2; EC 3.5.3.18). In fact, ADMA, total dimethylarginine (total DMA) and arginine were the top discriminant metabolites (according to their VIP values and their loadings, Fig. 6b, c) with a clearly increased concentrations 1 day after LS (LS_1 group) compared to controls or LS_7 groups. We hypothesize here that ADMA contributes to the preconditioning response by preventing NO levels from increasing. Although accelerated proteolysis is expected to occur 1 day after LS and thus cause an increase in amino acid derivatives such as ADMA, the increase in ADMA concentration observed here probably also came from the inhibition of DDAH by LS. Indeed, under basal (non-stressful) conditions, DDAH is not saturated thereby leading to limited ADMA accumulation despite the degradation of ADMA-containing proteins (Teerlink 2005). In addition, DDAH is very sensitive to oxidative stress such as that occurring upon LS, and DDAH inhibition has been effectively shown to lead to an increase in ADMA content (Murray-Rust et al. 2001). Aside ADMA, arginine itself accumulated 1 day after LS, likely due to proteolysis but also to decreased arginine consumption by ADMA-mediated NOS inhibition (Teerlink 2005).

As stated above, LS also caused an increase in phosphatidylcholines (PC), mainly unsaturated PCs (Fig. 6b), and this effect was observed 1 day after LS and remained visible 7 day after LS as compared to controls. In fact, the metabolite that correlated the most with LS (regardless of time) was PC aa 36:6 (Fig. 6b). 36:6 denotes a PUFA formed by the sum of two FAs: here, probably docosahexaenoic acid (DHA 22:6 (n-3), the most prevalent very long chain PUFA (VLCPUFA) in the photoreceptor membrane) and myristic acid (14:0). DHA and other PUFAs have a very important role in photoreceptor membrane and physiology and have been demonstrated to have neuroprotective effects on the retina (SanGiovanni and Chew 2005 but see Tanito et al. 2009).

Other important changes in the retina metabolome were found 7 days after LS. An increased concentration in phenylethylamine (PEA, a catabolic product of phenylalanine), along with decreased concentration in aromatic amino acids (tryptophan, phenylalanine and tyrosine), was typically found 7 days after LS and differentiated LS_1 and LS_7 groups. These changes suggest an increase in aromatic amino acid decarboxylase (AADC; EC 4.1.1.28) activity after LS to restore aromatic amino acid homeostasis in the retina. Accordingly, LIF and CNTF have been shown to inhibit AADC, corroborating the hypothesis of metabolic alterations being driven by neurotrophic factors released during LS (Berry et al. 1996; Chireux et al. 1994).

## Conclusion and perspectives

Using a wide-spectrum targeted metabolomics analysis, we have provided evidence for metabolic sexual dimorphism in retina and effects of mild LS. Sexual dimorphism was mostly visible in lipids. The metabolic effects of LS were remarkable in that they involved rather different aspects of retinal metabolism (amino acids, biogenic amines and lipids) and some changes persisted for one week after LS, indicating that this insult caused a considerable re-orchestration of primary carbon metabolism. The sample size in our study did not allow the investigation of the interaction between sex and LS, thus further investigations are needed to reveal a potential sexual dimorphism in retinal response to LS. Further, the limited sample size also hampered the use of a test set to carry out external validation and evaluate the risk of over-fitting in multivariate statistical analysis.

We also recognize that the retina is a highly differentiated tissue, containing multiple types of neuronal and non-neuronal cells arranged in layers. In order to assess which ones of these cell types are mostly responsible for the metabolomics changes observed here, both endo- and exo-metabolome analyses in cultured retinal cells (e.g., ARPE 19 in the case of RPE cells) under different hormonal and light treatments have to be undertaken. Further “omics” studies will also help shedding light on pathways effectively involved in metabolomics changes. Recently, methods for isotopic ^13^C labeling in cultured retina and subsequent fractionation into cell layers for mass spectrometry analysis have been published (Du et al. 2015). The potential of this technique is considerable since, in principle, it makes retinal fluxomics analyses feasible to assess metabolic reorganization. This will be addressed in a future study.

## Electronic supplementary material

Below is the link to the electronic supplementary material.

Supplementary material 1 (PDF 690 KB)

## Abbreviations

F: Female
GCL: Ganglion cell layer
INL: Inner nuclear layer
LC-MS/MS: Liquid chromatography-tandem mass spectrometry
LS: Light stress
LS_1 group: Group of animals whose retinas have been collected 1 day after commencement of LS
LS_7 group: Group of animals whose retinas have been collected 7 day after LS
Lyso-PC: Lysophosphatidylcholine
M: Male
NO: Nitric oxide
NOS: Nitric oxide synthase
ONL: Outer nuclear layer
OPLS-DA: Orthogonal partial least squares-discriminant analysis
PC: Phosphatidylcholine
PCA: Principal component analysis
PUFA: Polyunsaturated fatty acid
ROS: Rod outer segments
SM: Sphingomyelin
VIP: Variable importance in the projection
