# Supplementary material for: Retinal metabolic events in preconditioning light stress as revealed by wide-spectrum targeted metabolomics
Source: Metabolomics. 2017 Jan 20;13(3):22. doi: 10.1007/s11306-016-1156-9 (PMC5486622; doi:10.1007/s11306-016-1156-9)
Supplement: Supplementary file 1 — Supplementary material 1 (PDF 690 KB) [file 11306_2016_1156_MOESM1_ESM.pdf]

Supplementary Material

- Supplementary Text (and references)

- Supplementary Methods: S1

- Supplementary Tables: S2, S3, S4, S5, S6

Supplementary Text - *Differences between male and female retinal metabolome*

While retinal neurons, as most other CNS structures, rely on glucose as the main source of ATP, some retinal cellular types such as retinal pigment epithelium (RPE) cells, involve FA oxidation (FAO) and thus carnitine metabolism (Adijanto et al. 2014; Tini 2002). Indeed, shortly after light onset, RPE cells phagocyte shed tips of photoreceptors leading to the formation of disk-enriched phagosomes. Phagosomes eventually become phagolysosomes after having fused with early and then late endosomes (Kevany and Palczewski 2010). Before entering the mitochondria for oxidation, free fatty acids (FFA) resulting from lipid digestion in phagolysosomes are rapidly combined to coenzyme A, thereby forming acyl-CoA. FFA transport into the mitochondrial matrix requires bonding to carnitine catalyzed by carnitine palmitoyl-transferase 1 (CPT-1; EC 2.3.1.21). Acyl-carnitine is then transported into the mitochondrial matrix by the mitochondrial inner membrane transporter carnitine acyl-carnitine translocase (CACT) (Schooneman et al. 2013). Because of its role in FFA catabolism, CPT-1 is believed to be a key enzyme regulating the rate of FA oxidation (McGarry and Brown 1997). Once in the mitochondrial matrix, CPT-2 (EC 2.3.1.21) cleaves acyl-carnitine into carnitine and acyl-CoA that can undergo FAO (Ramsay et al. 2001). Evidence has been provided for carnitine uptake and oxidation of FA released from phagocytized photoreceptors by RPE cells (Adijanto et al. 2014; Tini 2002). Our results suggest a relatively decreased fatty acid (FA) oxidation rate in male compared to female retinas. That is, FA released from phagocytized photoreceptors in RPE cells may have outpaced mitochondrial oxidative capacity, eventually leading to an increased acyl carnitine accumulation in male retinas. Nevertheless, the activity of CPT-1, as measured by the (C16+C18)-to-C0 ratio, was not significantly higher in female compared to male retinas. It is possible that other "post-CPT-1 events" such as an increased mitochondria number or overall

mitochondrial activity could have led to an increase FAO capacity in females, a pattern similar to that found in human skeletal muscle (Kim et al. 2000).

As a matter of fact, a likely cause of the sexual dimorphism in lipid metabolism is the increased estrogen-mediated mitochondrial biogenesis in female retinas. Estrogens increase mitochondrial biogenesis by upregulating nuclear and mitochondrial transcription factors like the Nuclear Respiratory Factor 1 and 2 (NRF 1 and NRF 2), the mitochondrial transcription factor (Tfam) and the three members of the PPARγ coactivator 1 (PGC-1) family, PGC-1α, PGC-1β and PGC-1 related coactivator (PRC) (Klinge 2009; Mattingly et al. 2008; Tcherepanova et al. 2000). The interaction between PGC-1α and members of the peroxisome proliferator-activated receptor (PPARs) family has been shown to increase not only mitochondria biogenesis but also FAO in muscle and liver (Finck and Kelly 2006). It has also been shown that PPARγ expression in RPE cells is selectively enhanced after photoreceptor phagocytosis (Ershov and Bazan 2000). In addition, the peak in PGC-1α expression in mouse RPE cells has been found to coincide with maximal RPE phagocytosis activity (Stone et al. 2008). Recently, Iavovelli *et al*. have demonstrated that PGC-1α overexpression lead to a potent induction of mitochondrial respiration and fatty-acid oxidation in RPE cells (Iacovelli et al. 2016).

The expression of both subtypes of estrogen receptors alpha and beta (ER α and ER β) and androgen receptor (AR) in rat and human retinas has been well documented (Kobayashi et al. 1998; Munaut et al. 2001; Ogueta et al. 1999; Rocha 2000; Wickham et al. 2000). Furthermore, the retina, as well as other structures of the central nervous system (CNS), is capable of steroidogenesis with synthesis of estradiol (E2) from cholesterol and from testosterone aromatization (reviewed in (Cascio et al. 2015)). Taken as a whole, the hypothesis of a stimulation of mitochondria biogenesis and FAO capacity by estrogens in females is likely. We nevertheless recognize that further research has to be conducted to examine the specific involvement of estrogens in facilitating lipid recycling after photoreceptor phagocytosis.

In addition to lipids, some nitrogen-containing compounds have been found to be sexually dimorphic. In fact, male retinas had a relatively increased concentration in acetyl-ornithine (Ac-Orn) and tyrosine (**Fig. 4**). Deacetylation of Ac-Orn by aminoacylase 1 (EC 3.5.1.14) is the sole reaction of Ac-Orn metabolism mentioned in rat metabolic pathway database (e.g., KEGG, http://www.kegg.jp). Unfortunately, literature data are lacking about normal concentration and possible functions of this metabolite in the retina. Considering its

connections to glutamate metabolism and the urea cycle, one may assume a role of Ac-Orn in retina signaling but this needs further investigation.

Retinal tyrosine concentrations have been shown to parallel tyrosine plasmatic concentration (Fernstrom and Fernstrom 2007) and plasmatic tyrosine concentration has been found to be significantly higher in male rats compared to females (Chance et al. 1995). Armstrong *et al*. also found significantly higher tyrosine concentration in men compared to women plasma (Armstrong and Stave 1973). Thus, the higher content in retinal tyrosine in males is simply due to the larger availability in plasmatic tyrosine. It should be noted that tyrosine can be converted to dopamine by tyrosine hydroxylase (EC 1.14.16.2) in retinal dopaminergic neurons, and this reaction is stimulated by light and follows a circadian rhythm (Iuvone et al. 1978). Here, rat culling was carried out in the dark and that could explain why, unlike tyrosine, dopamine was not amongst top metabolites increased in male retinas.

References for Supplementary Text

Adijanto, J., Du, J., Moffat, C., Seifert, E. L., Hurley, J. B., & Philp, N. J. (2014). The retinal pigment epithelium utilizes fatty acids for ketogenesis implications for metabolic coupling with the outer retina. *Journal of Biological Chemistry*, *289*(30), 20570–20582. doi:10.1074/jbc.M114.565457

Armstrong, M. D., & Stave, U. (1973). A study of plasma free amino acid levels. II. Normal values for children and adults. *Metabolism*, *22*(4), 561–569. doi:10.1016/0026-0495(73)90069-3

Cascio, C., Deidda, I., Russo, D., & Guarneri, P. (2015). The estrogenic retina: The potential contribution to healthy aging and age-related neurodegenerative diseases of the retina. *Steroids*, *103*, 31–41. doi:10.1016/j.steroids.2015.08.002

Chance, W. T., Grossman, C. J., Newrock, R., Bovin, G., Yerian, S., Schmitt, G., & Mendenhall, C. (1995). Effects of electromagnetic fields and gender on neurotransmitters and amino acids in rats. *Physiology and Behavior*, *58*(4), 743–748. doi:10.1016/0031-9384(95)00090-6

Ershov, A. V, & Bazan, N. G. (2000). Photoreceptor phagocytosis selectively activates PPAR γ expression in retinal pigment epithelial cells. *Journal of Neuroscience Research*, *60*(3), 328–337. doi:10.1002/(SICI)1097-4547(20000501)60:3<328::AID-JNR7>3.0.CO;2-5

Fernstrom, J. D., & Fernstrom, M. H. (2007). Tyrosine, phenylalanine, and catecholamine synthesis and function in the brain. *The Journal of nutrition*, *137*(6 Suppl 1), 1539S–1548S. doi:137/6/1539S [pii]

Finck, B. N., & Kelly, D. P. (2006). PGC-1 coactivators: Inducible regulators of energy metabolism in health and disease. *Journal of Clinical Investigation*, *116*(3), 615–622. doi:10.1172/JCI27794

Iacovelli, J., Rowe, G. C., Khadka, A., Diaz-Aguilar, D., Spencer, C., Arany, Z., & Saint-Geniez, M. (2016). PGC-1α induces human RPE oxidative metabolism and antioxidant capacity. *Investigative Ophthalmology and Visual Science*, *57*(3), 1038–1051. doi:10.1167/iovs.15-17758

Iuvone, P. M., Galli, C. L., Garrison-Gund, C. K., & Neff, N. H. (1978). Light stimulates

tyrosine hydroxylase activity and dopamine synthesis in retinal amacrine neurons. *Science*, *202*(4370), 901–902. doi:10.1126/science.30997

Kevany, B. M., & Palczewski, K. (2010). Phagocytosis of Retinal Rod and Cone Photoreceptors. *Physiology (Bethesda, Md.)*, *25*(1), 8–15. doi:10.1152/physiol.00038.2009

Kim, J. Y., Hickner, R. C., Cortright, R. L., Dohm, G. L., & Houmard, J. a. (2000). Lipid oxidation is reduced in obese human skeletal muscle. *American journal of physiology. Endocrinology and metabolism*, *279*(5), E1039–E1044.

Klinge, C. M. (2009). Estrogenic control of mitochondrial function and biogenesis. *J Cell Biochem*, *105*(6), 1342–1351. doi:10.1002/jcb.21936

Kobayashi, K., Kobayashi, H., Ueda, M., & Honda, Y. (1998). Estrogen Receptor Expression in Bovine and Rat Retinas, *39*(11).

Mattingly, K. A., Ivanova, M. M., Riggs, K. A., Wickramasinghe, N. S., Barch, M. J., & Klinge, C. M. (2008). Estradiol stimulates transcription of nuclear respiratory factor-1 and increases mitochondrial biogenesis. *Molecular endocrinology (Baltimore, Md.)*, *22*(3), 609–22. doi:10.1210/me.2007-0029

McGarry, J. D., & Brown, N. F. (1997). The Mitochondrial Carnitine Palmitoyltransferase System - From Concept to Molecular Analysis. *European Journal of Biochemistry*, *244*(1), 1–14. doi:10.1111/j.1432-1033.1997.00001.x

Munaut, C., Lambert, V., Noël, A., Frankenne, F., Deprez, M., Foidart, J. M., & Rakic, J. M. (2001). Presence of oestrogen receptor type beta in human retina. *The British journal of ophthalmology*, *85*(7), 877–82. doi:10.1136/bjo.85.7.877

Ogueta, S. B., Schwartz, S. D., Yamashita, C. K., & Farber, D. B. (1999). Estrogen receptor in the human eye: in uence of gender and age on gene expression. *Invest. Ophthalmol. Vis. Sci.*, *40*(9), 1906–1911.

Ramsay, R. R., Gandour, R. D., & van der Leij, F. R. (2001). Molecular enzymology of carnitine transfer and transport. *Biochimica et Biophysica Acta (BBA) - Protein Structure and Molecular Enzymology*, *1546*(1), 21–43. doi:10.1016/S0167-4838(01)00147-9

Rocha, E. M. (2000). Identification of androgen receptor protein and 5alpha -reductase mRNA in human ocular tissues. *British Journal of Ophthalmology*, *84*(1), 76–84. doi:10.1136/bjo.84.1.76

Schooneman, M. G., Vaz, F. M., Houten, S. M., & Soeters, M. R. (2013). Acylcarnitines: Reflecting or inflicting insulin resistance? *Diabetes*, *62*(1), 1–8. doi:10.2337/db12-0466

Stone, J., van Driel, D., Valter, K., Rees, S., & Provis, J. (2008). The locations of mitochondria in mammalian photoreceptors: Relation to retinal vasculature. *Brain Research*, *1189*, 58–69. doi:10.1016/j.brainres.2007.10.083

Tcherepanova, I., Puigserver, P., Norris, J. D., Spiegelman, B. M., & McDonnell, D. P. (2000). Modulation of estrogen receptor-?? transcriptional activity by the coactivator PGC-1. *Journal of Biological Chemistry*, *275*(21), 16302–16308. doi:10.1074/jbc.M001364200

Tini, T. (2002). Mitochondrial Fatty Acid beta-Oxidation in the Retinal Pigment Epithelium. *Pediatric Research*, *52*(4), 595–600. doi:10.1203/01.PDR.0000030874.20888.2D

Wickham, L. A., Gao, J., Toda, I., Rocha, E. M., Ono, M., & Sullivan, D. A. (2000). Identification of androgen, estrogen and progesterone receptor mRNAs in the eye. *Acta ophthalmologica Scandinavica*, *78*(2), 146–153.

Supplementary Method S1. Tissue collection, histological and immunohistochemistry (IHC) analysis and terminal deoxynucleotidyl transferase dUTP nick end labelling (TUNEL) analysis.

Tissue collection

The superior aspect of each eye was marked for orientation. Each eye was enucleated with curved forceps and fixed in 4% paraformaldehyde. Fixed eyes were washed with 1M PBS and cryoprotected in 15% sucrose overnight at 4°C. All eyes were embedded in OCT medium (Leica Biosystems, Australia) prior to being frozen in acetone and dry ice. Cryosections were 12µm thick, cut in the parasagittal plane (superior-inferior) (CM1850; Leica); with the same orientation on Superfrost UltraPlus glass slides (ThermoScientific, Australia). Sections were then dried overnight at 37°C and stored at -20°C. Only sections containing the optic nerve head were used for analysis.

Retinas were visualized with the A1 Nikon confocal microscope and images were taken using NIS-Elements Advanced Research software (Nikon) at 10x magnification. Areas of interest were marked using ImageJ software (Rasband, W.S., ImageJ, U. S. National Institutes of Health, Bethesda, Maryland, USA) and their corrected total cell fluorescence (CTCF) was calculated as follows:

CTCF= Integrated density – (Area of interest x Mean fluorescence)

Histology

Cryosections were fixed with 10% neutral buffered formalin (Sigma-Aldrich, Cat# HT501128-4L) then hydrated sequentially through decreasing concentrations of ethanol and Milli-Q water (Millipore, USA). Sections were then stained with Harris' Haematoxylin for 10 minutes then

washed with tap water. After a brief rinse with Milli-Q water, the sections were counter-stained with Eosin-Y solution (Sigma-Aldrich, Cat# HT110216-500ML) for 1 minute. The sections were dehydrated through increasing concentrations of ethanol (70%, 90% and 100% ethanol). They were then cleared in xylene for 5 minutes and coverslipped with Micromount (Leica Biosystems, Australia) and clear nail varnish. Staining was visualised using the brightfield function of the A1 Nikon confocal microscope and images were taken using NIS-Elements Advanced Research software (Nikon) at 10x magnification.

Immunohistochemistry (IHC)

Cryosections were pre-treated with RevealIt for 3 hours at 37°C (ImmunoSolution, QLD, Australia). A hydrophobic barrier was marked using a PAP pen (Daido Sangyo, Tokyo, Japan) before incubation in 10% normal goat serum (NGS) (Sigma-Aldrich, Australia) for 1 hour at RT. Sections were then incubated in primary antibodies overnight at 4°C. To control for nonspecific binding by secondary antibodies, primary antibodies were omitted in designated negative control slides. All primary antibodies were diluted in 1% NGS. After the sections were washed in 0.1M PBS, there were incubated with secondary antibodies. Sections stained for COX5a were incubated with their secondary antibody, Mouse IgG-AlexFluor 594 conjugate (1:500; Life Technologies, Australia, Cat# A31623) for 2 hours at RT, while sections stained for IBA-1 were incubated with secondary antibody, Rabbit IgG-AlexFluor 488 conjugate (1:500; Life Technologies, Cat# A31627) for 4 hours at RT. All secondary antibodies were diluted with 0.1M PBS and the addition of all antibodies to slides were done in the dark. Sections were washed in 0.1M PBS before they were incubated in 0.05% Sudan Black B for 10 minutes to prevent auto-fluorescence. After another wash in 0.1M PBS, the blue fluorescent dye, bisbenzimide (BBZ)

(1:1000; Calbiochem, La Jolla, CA), was used to visualise cellular layers as it stains A-T rich regions. After a final wash in 0.1M PBS, sections were coverslipped with Aquamount (Polysciences, Warrington, PA, Cat# 18606) and clear nail varnish. Immunofluorescence was visualised with the A1 Nikon confocal microscope and images were taken using NIS-Elements Advanced Research software (Nikon) at 10x magnification.

Terminal deoxynucleotidyl transferase dUTP nick end labelling (TUNEL)

Cryosections used for the TUNEL assay were permeabilised using Triton (0.1% w/ PBS) for 2 minutes at RT. Following a wash in 0.1M PBS, the sections were placed in 1M TdT buffer for 10 minutes at RT. TUNEL reaction mixture was made up according to the manufacturer's instructions (contained fresh and contained 89.11% Milli-Q water (Millipore), 10.51% 10M TdT buffer, 0.25% biotin-dUTP and 0.13% terminal transferase). Sections were marked with a PAP pen before incubation with TUNEL reaction mixture for 1 hour at 37°C with humidity. The reaction was stopped using 2M saline sodium citrate buffer (SSC) for 15 minutes before incubation in 10% normal goat serum for 10 minutes at RT. Streptavidin-AlexFluor 594 conjugate (1:500; Life Technologies, Australia, Cat# S32356) was applied to sections under dim conditions and then incubated for 1 hour at 37°C with humidity. Sections were washed in 0.1M PBS before being stained with BBZ for 2 minutes to visualise cellular layers. After a final wash in 0.1M PBS, sections were coverslipped with Aquamount and clear nail varnish. In TUNEL and ONL thickness analyses, *n* = 6 animals were used per condition and at least 4 samples taken from each of the superior and inferior retina. Measurements were averaged for each of the superior and inferior retina for all samples and animals in the same experimental group.

Table S3: Metabolite concentrations (μmol/L of extract) in rat retina.

Sample ID	Protein (g/L)	CO	C12	C12:1	C14	C14:1	C14:2	C14:2-OH	C16
Control Female-1	1.69	75.077	0.061	0.089	0.173	0.043	0.015	0.014	0.467
Control Female-2	0.73	171.422	0.130	0.246	0.339	0.063	0.029	0.038	0.546
Control Female-3	2.11	120.529	0.078	0.123	0.174	0.037	0.018	0.022	0.370
Control Female-4	2.29	188.797	0.134	0.128	0.441	0.122	0.030	0.033	0.570
Control Female-5	0.79	86.005	0.076	0.077	0.292	0.062	0.018	0.017	0.684
Control Female-6	1.47	146.450	0.086	0.097	0.358	0.064	0.021	0.021	0.646
Control Male-1	1.07	216.173	0.134	0.103	0.354	0.123	0.038	0.022	0.912
Control Male-2	1.41	348.951	0.183	0.235	0.562	0.157	0.045	0.051	0.665
Control Male-3	2.55	159.890	0.148	0.054	0.324	0.167	0.046	0.018	0.676
Control Male-4	1.01	323.658	0.166	0.106	0.447	0.159	0.041	0.033	0.658
Control Male-5	1.59	139.412	0.110	0.045	0.279	0.150	0.044	0.016	0.580
Control Male-6	1.69	445.442	0.274	0.155	0.700	0.308	0.083	0.041	1.081
LS_1 Female-1	1.02	121.604	0.439	0.182	1.293	0.242	0.072	0.047	1.293
LS_1 Female-2	0.88	138.567	0.222	0.162	1.047	0.189	0.041	0.046	1.117
LS_1 Female-3	1.53	143.283	0.184	0.199	0.918	0.128	0.037	0.050	1.045
LS_1 Male-1	0.91	256.750	0.153	0.144	0.550	0.126	0.037	0.036	0.738
LS_1 Male-2	0.93	338.530	0.176	0.195	0.439	0.130	0.057	0.047	0.620
LS_1 Male-3	0.80	172.274	0.114	0.098	0.398	0.097	0.027	0.025	0.666
LS_7 Female-1	0.96	144.471	0.175	0.128	0.354	0.144	0.064	0.030	0.454
LS_7 Female-2	0.87	270.364	0.256	0.189	0.622	0.213	0.069	0.050	0.895
LS_7 Female-3	1.02	216.728	0.140	0.175	0.515	0.128	0.032	0.035	0.833
LS_7 Female-4	1.31	156.385	0.140	0.076	0.407	0.140	0.038	0.026	0.624
LS_7 Male-1	1.64	291.255	0.175	0.069	0.400	0.186	0.048	0.024	0.659
LS_7 Male-2	1.20	338.925	0.151	0.117	0.446	0.132	0.036	0.030	0.664

Table S3: Metabolite concentrations (µmol/L of extract) in rat retina.
(cont.)

Sample ID	C16-OH	C16:1	C16:2	C18	C18:1	C18:2	C2
Control Female-1	0.029	0.096	0.020	0.419	0.860	0.088	20.865
Control Female-2	0.070	0.119	0.036	0.514	0.589	0.198	64.379
Control Female-3	0.074	0.064	0.020	0.370	0.442	0.093	33.776
Control Female-4	0.101	0.142	0.050	0.543	0.580	0.196	46.452
Control Female-5	0.052	0.112	0.024	0.709	0.722	0.141	28.313
Control Female-6	0.036	0.107	0.032	0.584	0.688	0.183	47.433
Control Male-1	0.041	0.155	0.043	0.663	0.894	0.144	55.080
Control Male-2	0.093	0.151	0.058	0.484	0.574	0.188	94.244
Control Male-3	0.032	0.149	0.035	0.710	1.140	0.112	44.135
Control Male-4	0.086	0.134	0.057	0.568	0.670	0.202	79.670
Control Male-5	0.031	0.143	0.029	0.771	1.692	0.110	32.229
Control Male-6	0.137	0.211	0.085	1.062	1.101	0.266	92.065
LS_1 Female-1	0.091	0.153	0.073	2.191	0.569	0.218	42.397
LS_1 Female-2	0.180	0.172	0.068	1.472	0.599	0.226	40.174
LS_1 Female-3	0.117	0.145	0.052	1.632	0.597	0.173	44.355
LS_1 Male-1	0.067	0.112	0.053	1.053	0.650	0.178	63.253
LS_1 Male-2	0.067	0.115	0.055	0.635	0.701	0.190	80.472
LS_1 Male-3	0.041	0.114	0.042	0.816	0.698	0.169	42.808
LS_7 Female-1	0.053	0.117	0.045	0.533	0.449	0.130	49.204
LS_7 Female-2	0.085	0.205	0.065	0.862	0.910	0.291	88.273
LS_7 Female-3	0.115	0.152	0.058	0.747	0.717	0.251	63.541
LS_7 Female-4	0.048	0.124	0.041	0.584	0.571	0.160	44.703
LS_7 Male-1	0.070	0.131	0.046	0.702	0.617	0.162	68.997
LS_7 Male-2	0.054	0.128	0.047	0.565	0.630	0.162	80.526

Table S3: Metabolite concentrations (μmol/L of extract) in rat retina.
(cont.)

Sample ID	C3	C3-DC (C4-OH)	C3:1	C4	C4:1	C6 (C4:1-DC)	C5
Control Female-1	1.192	0.166	0.018	1.336	0.020	0.086	0.515
Control Female-2	2.933	0.537	0.034	2.946	0.057	0.130	1.295
Control Female-3	2.145	0.239	0.024	1.709	0.031	0.076	0.919
Control Female-4	3.112	0.433	0.036	2.972	0.045	0.178	1.449
Control Female-5	1.350	0.222	0.027	1.620	0.029	0.074	0.625
Control Female-6	1.761	0.417	0.024	2.063	0.042	0.095	0.878
Control Male-1	4.501	0.402	0.024	3.725	0.025	0.180	1.937
Control Male-2	5.907	0.728	0.033	6.072	0.047	0.271	3.228
Control Male-3	2.715	0.368	0.018	3.255	0.022	0.139	1.405
Control Male-4	4.581	0.662	0.028	4.403	0.040	0.203	2.675
Control Male-5	2.240	0.255	0.018	2.130	0.017	0.164	1.057
Control Male-6	4.911	0.751	0.033	6.220	0.039	0.367	3.611
LS_1 Female-1	2.695	0.332	0.026	3.276	0.041	0.305	1.764
LS_1 Female-2	2.052	0.390	0.028	2.847	0.054	0.216	1.515
LS_1 Female-3	2.454	0.384	0.032	2.878	0.037	0.223	1.582
LS_1 Male-1	4.082	0.464	0.029	4.968	0.038	0.196	2.745
LS_1 Male-2	5.141	0.694	0.034	6.964	0.042	0.241	3.750
LS_1 Male-3	2.643	0.363	0.024	4.020	0.022	0.146	1.925
LS_7 Female-1	2.397	0.363	0.032	2.596	0.049	0.195	1.204
LS_7 Female-2	4.830	0.629	0.047	4.980	0.069	0.278	2.403
LS_7 Female-3	4.147	0.505	0.031	3.389	0.066	0.191	1.960
LS_7 Female-4	2.388	0.329	0.028	2.426	0.046	0.166	1.396
LS_7 Male-1	5.001	0.482	0.027	4.976	0.036	0.232	3.419
LS_7 Male-2	4.921	0.625	0.034	4.847	0.034	0.201	3.423

Table S3: Metabolite concentrations (μmol/L of extract) in rat retina.
(cont.)

Sample ID	C5-OH (C3-DC-M)	C5:1	C5-DC (C6-OH)	C8	Ala	Arg	Asn
Control Female-1	0.237	0.039	0.069	0.096	403.758	169.360	49.324
Control Female-2	0.503	0.105	0.146	0.973	778.383	252.689	104.631
Control Female-3	0.347	0.061	0.101	0.471	555.661	156.620	62.852
Control Female-4	0.433	0.080	0.112	0.562	535.547	149.542	74.023
Control Female-5	0.284	0.047	0.096	0.113	484.013	129.321	64.033
Control Female-6	0.397	0.068	0.110	0.208	454.766	168.388	81.230
Control Male-1	0.557	0.076	0.071	0.263	518.814	183.599	88.838
Control Male-2	0.793	0.151	0.144	1.129	695.155	381.923	97.542
Control Male-3	0.416	0.069	0.069	0.096	356.677	79.945	62.915
Control Male-4	0.740	0.120	0.112	0.403	479.441	198.463	85.360
Control Male-5	0.323	0.047	0.053	0.087	261.593	66.181	27.452
Control Male-6	0.840	0.136	0.149	0.568	596.238	214.289	111.113
LS_1 Female-1	0.377	0.093	0.162	0.633	938.874	351.667	177.477
LS_1 Female-2	0.411	0.093	0.110	0.738	646.645	327.619	107.416
LS_1 Female-3	0.460	0.103	0.147	0.735	864.680	341.386	150.758
LS_1 Male-1	0.765	0.118	0.147	0.801	635.480	268.554	113.127
LS_1 Male-2	0.960	0.158	0.181	1.001	868.551	452.513	148.178
LS_1 Male-3	0.514	0.086	0.085	0.335	659.080	225.131	118.765
LS_7 Female-1	0.414	0.083	0.113	0.248	563.229	99.978	84.380
LS_7 Female-2	0.752	0.122	0.255	0.793	737.147	227.614	98.671
LS_7 Female-3	0.678	0.107	0.212	0.764	529.998	168.457	83.145
LS_7 Female-4	0.442	0.073	0.130	0.248	402.023	98.408	108.325
LS_7 Male-1	0.684	0.100	0.104	0.106	376.738	89.758	63.502
LS_7 Male-2	0.814	0.131	0.112	0.490	401.381	112.420	82.025

Table S3: Metabolite concentrations (μmol/L of extract) in rat retina.
(cont.)

Sample ID	Cit	Gly	His	Ile	Leu	Met	Orn
Control Female-1	17.765	580.539	47.578	95.593		50.197	13.488
Control Female-2	35.935	1333.280	96.504	215.865	534.583	39.618	41.522
Control Female-3	31.733	639.418	67.755	121.210	406.531	40.857	17.296
Control Female-4	31.591	1046.790	46.265	165.431	316.842	26.731	17.665
Control Female-5	18.833	1016.990	64.033	118.021	382.314	44.886	16.134
Control Female-6	24.725	990.169	68.185	116.804	332.626	53.066	24.606
Control Male-1	16.939	912.071	108.975	166.423		91.207	25.645
Control Male-2	32.560	1134.780	108.944	232.176	611.351	11.471	43.550
Control Male-3	13.529		37.796	87.514	262.068	31.032	9.461
Control Male-4	26.960	1337.310	69.427	140.133	364.916	47.588	27.529
Control Male-5	6.344	599.157	33.600	50.517	133.538	33.052	7.910
Control Male-6	26.786	1140.890	61.509	155.756	323.417	12.203	22.917
LS_1 Female-1	53.134	1457.060	127.082	230.063	737.296	27.169	42.288
LS_1 Female-2	38.240	1036.560	77.017	171.866	481.224	12.675	24.383
LS_1 Female-3	48.716	1198.590	104.908	189.382	485.915	73.012	31.148
LS_1 Male-1	24.003	1200.130	120.997	182.971	430.867	74.172	37.184
LS_1 Male-2	48.557	1208.220	89.591	218.847	582.453	69.986	39.324
LS_1 Male-3	25.515	1226.800	95.273	150.740		59.839	22.774
LS_7 Female-1	25.544	1109.710	67.525	118.299	240.785	20.205	15.494
LS_7 Female-2	25.303	1941.080	116.696	209.128	485.269	80.647	28.192
LS_7 Female-3	29.948	1714.120	73.687	146.784	318.196	53.591	24.431
LS_7 Female-4	32.345		54.620	108.325	227.330	13.579	16.630
LS_7 Male-1	19.906		45.551	79.988	147.154	24.424	10.319
LS_7 Male-2	26.398	1282.420	59.041	119.082	258.149	24.732	16.821

Table S3: Metabolite concentrations (μmol/L of extract) in rat retina.
(cont.)

Sample ID	Phe	Pro	Ser	Thr	Trp	Tyr	Val
Control Female-1	84.244	154.519	219.121	191.185	44.523	156.702	169.797
Control Female-2	152.375	316.179	344.114	394.906	76.061	184.120	307.290
Control Female-3	78.310	204.968	279.873	223.354	42.015	125.977	155.939
Control Female-4	74.397	185.993	373.855	243.006	45.517	132.718	184.123
Control Female-5	95.422	170.754	260.526	271.198	41.998	105.466	214.071
Control Female-6	113.840	173.131	308.316	144.079	42.749	148.822	170.167
Control Male-1	107.790	161.685	197.221	217.949	64.556	241.047	273.029
Control Male-2	163.485	320.101	417.642	295.372	60.860	266.522	287.129
Control Male-3	83.729	149.956	193.003	152.321	35.479	170.297	145.225
Control Male-4	118.793	194.195	247.545	190.638	54.275	230.473	194.195
Control Male-5	60.307	101.034	140.978	111.216	25.376	198.544	86.937
Control Male-6	123.017	299.607	415.680	244.051	49.207	213.296	250.995
LS_1 Female-1	135.846	422.877	457.934	370.291	71.758	225.680	318.801
LS_1 Female-2	110.639	369.511	380.253	286.801	65.309	148.234	244.909
LS_1 Female-3	134.561	416.143	647.887	322.697	79.117	218.039	277.844
LS_1 Male-1	126.899	288.228	357.088	220.352	66.401	207.564	257.733
LS_1 Male-2	145.898	372.725	479.869	279.259	59.613	234.805	322.572
LS_1 Male-3	128.553	237.530	649.944	195.766	49.855	258.411	239.487
LS_7 Female-1	92.650	213.566	539.150	183.206	39.363	139.237	187.394
LS_7 Female-2	119.006	377.817	451.762	317.736	65.049	143.270	306.182
LS_7 Female-3	120.185	278.791	330.017	241.356	59.009	122.156	215.743
LS_7 Female-4	77.048	152.571	306.667	175.456	32.879	116.716	144.179
LS_7 Male-1	68.997	152.649	176.462	132.499	29.187	115.403	130.668
LS_7 Male-2	106.591	169.046	209.018	162.384	45.301	181.537	192.363

Table S3: Metabolite concentrations (μmol/L of extract) in rat retina.
(cont.)

Sample ID	Ac-Orn	ADMA	c4-OH-Pro	Carnosine	Creatinine	Dopamine	Histamine
Control Female-1	1.152	0.594	17.547	0.089	21.170	0.066	0.252
Control Female-2	1.575	1.117	25.015	0.262	46.982	0.190	0.747
Control Female-3	0.688	0.695	19.407	0.185	29.485	0.119	0.406
Control Female-4	1.028	0.745	12.805	0.272	43.180	0.149	0.555
Control Female-5	1.180	0.684	12.932	0.145	26.994	0.099	0.383
Control Female-6	2.164	0.978	22.768	0.140	30.357	0.095	0.343
Control Male-1	4.223	0.588	23.749	0.158	23.039	0.102	0.376
Control Male-2	1.841	0.904	29.675	0.290	46.161	0.000	0.765
Control Male-3	2.564	0.465	14.854	0.099	33.634	0.076	0.286
Control Male-4	3.749	0.747	17.997	0.196	36.136	0.127	0.437
Control Male-5	2.428	0.207	9.203	0.128	22.204	0.080	0.240
Control Male-6	3.234	0.736	28.969	0.201	40.675	0.137	0.571
LS_1 Female-1	0.879	2.432	39.111	0.348	34.619	0.164	0.633
LS_1 Female-2	0.795	1.364	20.194	0.267	37.273	0.149	0.613
LS_1 Female-3	1.179	2.031	27.411	0.269	41.365	0.176	0.688
LS_1 Male-1	3.168	1.643	18.986	0.191	33.250	0.155	0.570
LS_1 Male-2	1.767	1.379	26.444	0.284	39.324	0.174	0.657
LS_1 Male-3	3.380	1.194	11.877	0.155	26.167	0.104	0.380
LS_7 Female-1	1.057	0.516	15.285	0.394	32.663	0.181	0.762
LS_7 Female-2	1.583	1.130	28.654	0.255	51.069	0.267	0.757
LS_7 Female-3	1.162	0.945	16.944	0.204	43.444	0.156	0.577
LS_7 Female-4	1.533	0.564	9.917	0.159	32.421	0.134	0.468
LS_7 Male-1	2.155	0.609	16.303	0.138	42.742	0.125	0.374
LS_7 Male-2	2.581	0.366	17.654	0.177	33.559	0.157	0.470

Table S3: Metabolite concentrations (μmol/L of extract) in rat retina.
(cont.)

Sample ID	Kynurenine	Met-SO	PEA	Putrescine	SDMA	Serotonin	Spermidine
Control Female-1	0.243	3.322	0.015	2.017	1.131	0.017	8.424
Control Female-2	0.340	37.586	0.050	10.222	1.333	0.052	19.428
Control Female-3	0.466	7.422	0.035	3.956	0.851	0.031	12.462
Control Female-4	0.150	50.844	0.051	3.823	0.446	0.035	13.085
Control Female-5	0.129	10.044	0.030	0.414	0.747	0.020	10.358
Control Female-6	0.176	10.495	0.027	3.018	0.493	0.000	11.562
Control Male-1	0.187	5.911	0.036	3.163	0.888	0.024	11.549
Control Male-2	0.297	75.011	0.016	9.988	0.606	0.052	14.563
Control Male-3	0.099	15.327	0.075	0.000	0.081	0.019	2.422
Control Male-4	0.300	16.290	0.037	3.841	0.825	0.025	11.310
Control Male-5	0.134	5.757	0.065	0.085	0.105	0.013	2.228
Control Male-6	0.261		0.032	3.135	0.455	0.035	12.203
LS_1 Female-1	0.451	78.112	0.043	3.604	0.784	0.046	8.940
LS_1 Female-2	0.202	58.649	0.020	8.121	0.842	0.048	11.064
LS_1 Female-3	0.320	21.181	0.032	8.771	1.458	0.044	12.958
LS_1 Male-1	0.205	12.690	0.052	8.047	0.743	0.036	13.379
LS_1 Male-2	0.269	23.936	0.041	9.198	1.091	0.046	16.642
LS_1 Male-3	0.135	11.289	0.039	4.222	0.446	0.023	11.942
LS_7 Female-1	0.183	43.132	0.137	2.083	0.389	0.046	6.585
LS_7 Female-2	0.403	17.793	0.106	9.393	0.653	0.046	15.482
LS_7 Female-3	0.221	13.890	0.068	6.462	0.911	0.037	15.565
LS_7 Female-4	0.114		0.023	1.404	0.349	0.027	8.239
LS_7 Male-1	0.079	18.990	0.054	0.000	0.562	0.023	4.128
LS_7 Male-2	0.302	32.394	0.022	3.339	0.594	0.028	12.325

Table S3: Metabolite concentrations (μmol/L of extract) in rat retina.

(cont.)

Sample ID	Spermine	t4-OH-Pro	Taurine	total DMA	lysoPC a C16:0	lysoPC a C16:1	lysoPC a C17:0
Control Female-1	14.928	16.718	44.086	1.458	10.345	0.624	0.108
Control Female-2	47.109	20.698	135.868	1.486	27.682	1.030	0.342
Control Female-3	31.596	15.185	67.347	1.103	13.006	0.673	0.159
Control Female-4	26.544	11.496	108.418	0.807	20.749	0.821	0.255
Control Female-5	22.977	13.309	99.816	0.785	15.255	0.753	0.175
Control Female-6	26.148	17.728	56.505	1.109	19.151	0.777	0.224
Control Male-1	18.715	19.900	59.225	1.338	13.918	0.776	0.179
Control Male-2	27.889	34.620	186.840	1.539	37.368	1.116	0.598
Control Male-3	3.255	12.015	52.035	0.639	13.482	0.539	0.170
Control Male-4	26.391	21.411	70.636	0.854	23.616	0.839	0.317
Control Male-5	4.503	8.380	66.965	0.326	8.733	0.384	0.121
Control Male-6	26.885	25.794	157.740	1.111	25.596	0.954	0.320
LS_1 Female-1	15.118	34.838	83.261	3.133	24.211	0.803	0.353
LS_1 Female-2	14.179	20.302	272.837	2.148	24.813	0.930	0.345
LS_1 Female-3	24.047	24.047	175.677	3.551	26.289	1.079	0.328
LS_1 Male-1	33.053	17.117	156.411	1.928	25.085	0.913	0.326
LS_1 Male-2	36.589	20.175	120.822	2.131	29.180	1.067	0.337
LS_1 Male-3	28.060	10.702	77.001	1.638	18.337	0.816	0.243
LS_7 Female-1	12.039	15.285	258.583	0.578	20.624	0.550	0.320
LS_7 Female-2	25.188	27.961	129.405	1.363	31.427	1.133	0.404
LS_7 Female-3	38.420	14.481	136.933	1.034	25.712	1.025	0.273
LS_7 Female-4	21.665	8.468	127.396	0.632	18.919	0.701	0.186
LS_7 Male-1	7.083	17.341	94.643	0.684	17.952	0.518	0.218
LS_7 Male-2	29.562	22.734	119.915	0.660	22.401	0.796	0.306

Table S3: Metabolite concentrations (μmol/L of extract) in rat retina.
(cont.)

Sample ID	lysoPC a C18:0	lysoPC a C18:1	lysoPC a C18:2	lysoPC a C20:3	lysoPC a C20:4	lysoPC a C24:0	lysoPC a C26:0
Control Female-1	4.496	5.413	0.284	0.110	1.672	0.115	0.111
Control Female-2	15.492	12.025	0.978	0.223	4.698	0.395	0.291
Control Female-3	6.455	7.014	0.449	0.150	2.492	0.185	0.147
Control Female-4	13.178	8.907	0.609	0.195	2.963	0.303	0.294
Control Female-5	7.973	6.968	0.486	0.123	2.090	0.214	0.206
Control Female-6	8.716	8.419	0.694	0.187	3.386	0.209	0.185
Control Male-1	6.989	6.515	0.467	0.147	2.268	0.288	0.245
Control Male-2	26.790	12.832	1.158	0.279	4.630	0.475	0.468
Control Male-3	7.096	6.055	0.343	0.118	2.238	0.322	0.375
Control Male-4	13.089	9.888	0.768	0.203	4.232	0.351	0.353
Control Male-5	4.034	3.697	0.230	0.095	1.234	0.268	0.278
Control Male-6	15.476	10.318	0.670	0.239	3.790	0.389	0.420
LS_1 Female-1	18.076	8.589	1.621	0.213	3.703	0.438	0.298
LS_1 Female-2	16.005	9.582	1.364	0.209	3.727	0.323	0.256
LS_1 Female-3	16.696	11.899	1.123	0.245	4.498	0.358	0.248
LS_1 Male-1	13.575	10.526	0.801	0.223	4.515	0.459	0.292
LS_1 Male-2	16.186	12.310	0.921	0.276	5.255	0.303	0.270
LS_1 Male-3	10.245	8.353	0.563	0.193	3.374	0.232	0.216
LS_7 Female-1	14.238	8.354	1.029	0.164	3.162	0.561	0.355
LS_7 Female-2	18.833	13.634	1.068	0.267	4.518	0.553	0.380
LS_7 Female-3	13.201	11.526	0.745	0.209	4.354	0.414	0.313
LS_7 Female-4	9.917	8.620	0.507	0.128	2.601	0.198	0.167
LS_7 Male-1	8.304	7.449	0.598	0.151	2.827	0.332	0.345
LS_7 Male-2	10.576	9.493	0.600	0.207	3.889	0.312	0.301

Table S3: Metabolite concentrations (μmol/L of extract) in rat retina.
(cont.)

Sample ID	lysoPC a C26:1	lysoPC a C28:0	lysoPC a C28:1	PC aa C24:0	PC aa C28:1	PC aa C30:0	PC aa C30:2
Control Female-1	0.048	0.109	0.058	0.042	0.182	6.766	0.089
Control Female-2	0.105	0.235	0.132	0.118	0.282	10.412	0.117
Control Female-3	0.061	0.161	0.089	0.069	0.195	7.422	0.073
Control Female-4	0.102	0.221	0.141	0.115	0.246	9.057	0.105
Control Female-5	0.075	0.187	0.080	0.073	0.306	9.354	0.156
Control Female-6	0.058	0.143	0.069	0.065	0.212	7.767	0.097
Control Male-1	0.096	0.173	0.113	0.080	0.251	8.114	0.159
Control Male-2	0.188	0.352	0.214	0.179	0.301	6.677	0.088
Control Male-3	0.109	0.180	0.135	0.089	0.325	7.096	0.150
Control Male-4	0.146	0.256	0.149	0.109	0.272	10.599	0.095
Control Male-5	0.089	0.150	0.085	0.072	0.318	9.868	0.147
Control Male-6	0.136	0.274	0.157	0.140	0.375	10.417	0.210
LS_1 Female-1	0.128	0.252	0.138	0.142	0.203	7.077	0.073
LS_1 Female-2	0.118	0.257	0.134	0.125	0.215	7.079	0.085
LS_1 Female-3	0.111	0.247	0.143	0.120	0.247	11.052	0.071
LS_1 Male-1	0.121	0.249	0.138	0.124	0.285	12.592	0.131
LS_1 Male-2	0.105	0.242	0.166	0.121	0.242	11.968	0.123
LS_1 Male-3	0.080	0.170	0.113	0.078	0.245	11.420	0.147
LS_7 Female-1	0.181	0.231	0.194	0.183	0.364	12.353	0.144
LS_7 Female-2	0.170	0.370	0.205	0.207	0.343	13.172	0.132
LS_7 Female-3	0.117	0.264	0.157	0.150	0.313	13.398	0.117
LS_7 Female-4	0.073	0.149	0.083	0.076	0.240	7.781	0.089
LS_7 Male-1	0.112	0.170	0.117	0.102	0.227	6.900	0.047
LS_7 Male-2	0.134	0.240	0.170	0.109	0.306	10.742	0.127

Table S3: Metabolite concentrations (µmol/L of extract) in rat retina.
(cont.)

Sample ID	PC aa C32:0	PC aa C32:1	PC aa C32:2	PC aa C32:3	PC aa C34:1	PC aa C34:2	PC aa C34:3
Control Female-1	95.156	14.361	1.235	0.142	109.560	8.206	0.650
Control Female-2	273.006	27.555	1.803	0.330	306.020	18.920	1.676
Control Female-3	162.068	18.045	1.566	0.185	186.582	13.143	1.008
Control Female-4	222.444	26.263	1.757	0.297	257.025	18.225	1.795
Control Female-5	135.599	23.039	2.825	0.276	168.871	15.757	1.312
Control Female-6	161.273	20.218	1.660	0.247	187.361	14.052	1.316
Control Male-1	150.432	21.084	2.162	0.200	171.161	12.970	0.971
Control Male-2	177.223	18.959	1.594	0.280	228.055	14.288	1.203
Control Male-3	90.352	17.976	1.604	0.243	105.489	8.515	0.984
Control Male-4	280.978	28.382	1.871	0.320	301.607	21.198	1.842
Control Male-5	98.293	21.773	1.531	0.252	115.524	11.239	1.132
Control Male-6	202.384	30.258	3.512	0.418	261.908	20.338	1.964
LS_1 Female-1	154.471	18.843	1.468	0.302	185.146	13.366	1.369
LS_1 Female-2	168.643	19.979	1.396	0.255	207.313	16.327	1.536
LS_1 Female-3	232.990	28.158	2.018	0.339	289.057	19.686	1.744
LS_1 Male-1	313.805	33.446	2.204	0.522	351.186	23.511	2.479
LS_1 Male-2	327.131	30.661	1.869	0.423	368.165	22.683	2.314
LS_1 Male-3	229.699	29.300	2.486	0.374	259.064	21.534	2.029
LS_7 Female-1	206.238	37.479	1.403	0.530	308.833	27.533	2.680
LS_7 Female-2	313.114	37.666	2.299	0.426	388.215	25.534	2.184
LS_7 Female-3	333.958	37.435	2.256	0.451	395.036	26.795	2.532
LS_7 Female-4	106.037	24.411	2.105	0.350	167.828	16.020	1.785
LS_7 Male-1	97.696	22.836	0.837	0.315	156.313	13.677	1.417
LS_7 Male-2	234.833	30.229	2.273	0.342	284.797	19.986	1.974

Table S3: Metabolite concentrations (μmol/L of extract) in rat retina.

(cont.)

Sample ID	PC aa C34:4	PC aa C36:0	PC aa C36:1	PC aa C36:2	PC aa C36:3	PC aa C36:4	PC aa C36:5
Control Female-1	0.168	1.266	48.451	13.662	2.043	22.436	0.685
Control Female-2	0.535	3.441	135.868	34.919	5.168	72.632	2.324
Control Female-3	0.238	2.172	88.524	22.948	3.228	40.857	1.137
Control Female-4	0.479	2.589	115.895	31.404	5.346	55.704	2.467
Control Female-5	0.335	1.808	71.566	22.914	3.823	37.918	1.475
Control Female-6	0.348	1.820	84.194	22.887	3.706	43.224	1.376
Control Male-1	0.258	1.635	76.401	21.262	3.246	37.667	1.031
Control Male-2	0.412	1.758	85.589	25.004	4.410	50.419	1.470
Control Male-3	0.373	0.462	23.889	10.123	2.351	41.344	1.509
Control Male-4	0.485	3.685	147.247	39.337	6.516	76.824	2.155
Control Male-5	0.338	0.474	31.289	13.863	3.133	39.944	1.308
Control Male-6	0.568	2.093	106.152	31.548	5.655	66.172	2.302
LS_1 Female-1	0.458	1.654	74.935	20.377	3.462	46.670	2.377
LS_1 Female-2	0.447	2.009	84.536	24.491	4.383	44.578	2.041
LS_1 Female-3	0.525	2.828	124.469	33.142	4.921	63.418	2.878
LS_1 Male-1	0.707	4.456	167.231	44.070	7.348	91.584	2.745
LS_1 Male-2	0.650	3.442	173.254	42.174	7.101	97.228	2.314
LS_1 Male-3	0.523	3.041	122.680	35.173	5.945	65.908	2.003
LS_7 Female-1	0.808	1.110	75.586	35.804	7.381	106.783	4.460
LS_7 Female-2	0.642	3.466	174.466	45.407	7.371	79.145	3.027
LS_7 Female-3	0.635	4.325	187.174	47.779	7.960	87.578	3.231
LS_7 Female-4	0.529	0.816	47.221	19.758	4.241	45.924	2.449
LS_7 Male-1	0.529	0.623	36.697	16.547	4.299	57.763	2.674
LS_7 Male-2	0.523	2.515	126.577	33.976	5.721	70.283	2.390

Table S3: Metabolite concentrations (μmol/L of extract) in rat retina.

(cont.)

Sample ID	PC aa C36:6	PC aa C38:0	PC aa C38:1	PC aa C38:3	PC aa C38:4	PC aa C38:5	PC aa C38:6
Control Female-1	0.291	0.299	0.581	1.043	26.757	4.889	38.193
Control Female-2	0.923	0.919	1.524	2.286	78.981	15.872	118.599
Control Female-3	0.439	0.547	1.062	1.621	48.688	8.989	67.755
Control Female-4	0.821	0.647	1.224	2.056	63.462	14.580	96.268
Control Female-5	0.634	0.519	0.835	1.632	38.859	9.354	61.082
Control Female-6	0.640	0.534	1.026	1.719	49.924	10.139	70.557
Control Male-1	0.368	0.416	0.912	1.587	44.005	8.647	56.027
Control Male-2	0.577	0.725	0.893	2.047	50.832	11.636	68.416
Control Male-3	0.591	0.299	0.274	0.447	24.835	8.941	62.915
Control Male-4	0.782	0.839	1.757	3.002	89.628	18.495	116.659
Control Male-5	0.517	0.294	0.308	0.862	30.741	10.495	60.699
Control Male-6	0.875	0.665	1.210	2.044	66.767	17.758	100.200
LS_1 Female-1	1.074	0.701	0.997	1.512	47.765	9.674	90.272
LS_1 Female-2	0.975	0.646	1.052	1.740	50.378	10.291	79.058
LS_1 Female-3	1.383	0.922	1.508	2.093	75.878	14.328	116.121
LS_1 Male-1	1.712	1.023	1.977	2.725	104.274	21.150	155.427
LS_1 Male-2	1.687	0.997	1.961	3.078	108.740	22.683	162.996
LS_1 Male-3	1.305	0.842	1.527	2.708	73.739	16.640	115.502
LS_7 Female-1	1.392	0.914	0.784	1.602	75.062	27.743	162.268
LS_7 Female-2	1.143	1.155	2.103	2.877	93.472	20.682	129.405
LS_7 Female-3	1.074	1.162	2.187	3.625	102.453	23.446	142.843
LS_7 Female-4	0.877	0.442	0.529	0.999	33.871	12.969	80.100
LS_7 Male-1	0.775	0.470	0.387	0.775	35.170	16.303	89.758
LS_7 Male-2	0.849	0.784	1.307	2.115	73.115	18.487	102.427

Table S3: Metabolite concentrations (μmol/L of extract) in rat retina.
(cont.)

Sample ID	PC aa C40:2	PC aa C40:3	PC aa C40:4	PC aa C40:5	PC aa C40:6	PC aa C42:0	PC aa C42:1
Control Female-1	0.137	0.062	2.086	1.641	103.886	0.042	0.038
Control Female-2	0.353	0.104	5.727	4.800	307.290	0.140	0.105
Control Female-3	0.204	0.112	3.691	3.779	176.368	0.076	0.058
Control Female-4	0.293	0.140	4.757	5.645	223.378	0.137	0.099
Control Female-5	0.208	0.090	2.894	2.593	126.810	0.080	0.068
Control Female-6	0.188	0.116	3.688	4.204	167.795	0.092	0.063
Control Male-1	0.252	0.147	3.400	3.719	152.209	0.094	0.082
Control Male-2	0.466	0.195	4.341	2.116	164.859	0.218	0.179
Control Male-3	0.191	0.125	1.504	1.348	78.526	0.117	0.101
Control Male-4	0.422	0.230	6.829	8.394	252.525	0.170	0.124
Control Male-5	0.176	0.126	1.809	2.260	81.454	0.105	0.092
Control Male-6	0.353	0.188	4.950	3.750	186.510	0.177	0.150
LS_1 Female-1	0.273	0.103	3.232	2.191	168.713	0.113	0.104
LS_1 Female-2	0.228	0.121	3.781	2.299	170.792	0.122	0.090
LS_1 Female-3	0.310	0.189	5.333	4.847	259.155	0.122	0.110
LS_1 Male-1	0.415	0.277	7.565	10.919	322.658	0.140	0.109
LS_1 Male-2	0.463	0.173	8.594	9.335	340.809	0.144	0.146
LS_1 Male-3	0.331	0.162	5.501	8.222	234.267	0.098	0.095
LS_7 Female-1	0.326	0.220	4.188	5.549	205.191	0.151	0.107
LS_7 Female-2	0.453	0.246	7.244	6.678	272.675	0.198	0.172
LS_7 Female-3	0.374	0.213	7.901	10.935	314.255	0.155	0.128
LS_7 Female-4	0.133	0.071	2.075	1.564	95.357	0.080	0.047
LS_7 Male-1	0.230	0.172	2.125	2.461	90.979	0.135	0.109
LS_7 Male-2	0.401	0.241	5.779	5.779	182.370	0.157	0.127

Table S3: Metabolite concentrations (μmol/L of extract) in rat retina.

(cont.)

Sample ID	PC aa C42:2	PC aa C42:4	PC aa C42:5	PC aa C42:6	PC ae C30:0	PC ae C30:1	PC ae C30:2
Control Female-1	0.082	0.293	0.321	1.323	0.203	0.104	0.036
Control Female-2	0.229	0.898	0.837	4.025	0.272	0.118	0.077
Control Female-3	0.129	0.506	0.537	2.247	0.204	0.093	0.045
Control Female-4	0.220	0.818	0.875	2.701	0.248	0.097	0.064
Control Female-5	0.144	0.402	0.374	1.482	0.291	0.169	0.057
Control Female-6	0.127	0.586	0.554	2.140	0.199	0.088	0.043
Control Male-1	0.190	0.634	0.567	2.179	0.261	0.117	0.059
Control Male-2	0.478	1.293	0.719	2.157	0.267	0.125	0.103
Control Male-3	0.237	0.497	0.314	0.766	0.228	0.102	0.078
Control Male-4	0.325	1.188	1.117	3.735	0.286	0.122	0.086
Control Male-5	0.219	0.501	0.347	0.869	0.262	0.118	0.072
Control Male-6	0.352	0.991	0.715	2.212	0.311	0.130	0.088
LS_1 Female-1	0.255	0.563	0.561	2.103	0.249	0.088	0.065
LS_1 Female-2	0.211	0.652	0.583	2.159	0.238	0.099	0.078
LS_1 Female-3	0.211	0.845	0.875	3.277	0.287	0.131	0.072
LS_1 Male-1	0.301	1.171	1.200	4.259	0.292	0.118	0.078
LS_1 Male-2	0.279	1.345	1.126	4.069	0.287	0.122	0.062
LS_1 Male-3	0.217	0.914	0.868	3.145	0.286	0.131	0.057
LS_7 Female-1	0.292	0.827	0.609	1.686	0.295	0.122	0.113
LS_7 Female-2	0.318	0.991	1.060	3.050	0.363	0.158	0.089
LS_7 Female-3	0.262	1.162	1.241	3.645	0.313	0.125	0.073
LS_7 Female-4	0.118	0.281	0.272	0.793	0.195	0.082	0.052
LS_7 Male-1	0.241	0.521	0.382	0.696	0.181	0.061	0.074
LS_7 Male-2	0.287	0.999	0.858	2.090	0.271	0.114	0.070

Table S3: Metabolite concentrations (μmol/L of extract) in rat retina.
(cont.)

Sample ID	PC ae C32:1	PC ae C32:2	PC ae C34:0	PC ae C34:1	PC ae C34:2	PC ae C34:3	PC ae C36:0
Control Female-1	0.860	0.224	1.733	2.169	0.842	0.109	0.441
Control Female-2	1.422	0.348	5.270	5.536	1.790	0.231	1.473
Control Female-3	1.076	0.249	3.480	3.493	1.233	0.148	0.885
Control Female-4	1.308	0.321	4.187	4.767	1.570	0.201	1.131
Control Female-5	1.431	0.412	2.630	3.760	1.337	0.204	0.835
Control Female-6	1.049	0.261	3.172	3.552	1.014	0.149	0.937
Control Male-1	1.179	0.311	3.074	3.548	1.262	0.175	0.859
Control Male-2	1.015	0.330	3.490	3.943	1.415	0.174	2.129
Control Male-3	0.681	0.219	1.334	2.044	0.425	0.110	0.606
Control Male-4	1.914	0.388	5.947	6.246	2.042	0.254	1.501
Control Male-5	0.936	0.247	1.723	2.498	0.552	0.125	0.521
Control Male-6	1.548	0.426	3.740	4.841	1.657	0.221	1.111
LS_1 Female-1	0.885	0.275	2.684	3.078	1.117	0.185	0.915
LS_1 Female-2	0.981	0.264	2.911	3.620	1.225	0.188	1.407
LS_1 Female-3	1.508	0.350	4.149	5.021	1.844	0.229	1.420
LS_1 Male-1	1.751	0.385	6.374	6.384	2.154	0.253	1.515
LS_1 Male-2	1.676	0.375	6.315	6.736	2.018	0.250	1.744
LS_1 Male-3	1.768	0.401	5.077	5.697	1.951	0.218	1.377
LS_7 Female-1	1.141	0.289	3.224	5.884	1.078	0.225	1.487
LS_7 Female-2	2.010	0.477	6.031	7.048	2.392	0.312	1.629
LS_7 Female-3	2.256	0.441	7.024	7.792	2.473	0.298	2.148
LS_7 Female-4	0.786	0.241	1.579	2.670	0.778	0.135	0.602
LS_7 Male-1	0.629	0.177	1.545	2.797	0.557	0.116	0.928
LS_7 Male-2	1.507	0.341	4.547	5.396	1.407	0.230	2.307

Table S3: Metabolite concentrations (µmol/L of extract) in rat retina.
(cont.)

Sample ID	PC ae C36:1	PC ae C36:2	PC ae C36:3	PC ae C36:4	PC ae C36:5	PC ae C38:0	PC ae C38:1
Control Female-1	1.165	0.502	0.375	0.340	0.738	0.559	0.197
Control Female-2	3.581	1.270	0.703	1.032	2.362	1.892	0.649
Control Female-3	2.377	0.865	0.489	0.541	1.382	0.953	0.374
Control Female-4	2.981	1.075	0.697	0.838	2.122	1.626	0.544
Control Female-5	2.084	0.904	0.554	0.613	1.281	1.212	0.391
Control Female-6	2.277	0.795	0.474	0.611	1.204	1.269	0.393
Control Male-1	2.251	0.859	0.490	0.555	1.338	0.841	0.387
Control Male-2	3.036	1.048	0.555	0.875	1.855	1.704	0.820
Control Male-3	1.168	0.386	0.214	0.434	0.441	1.254	0.278
Control Male-4	4.076	1.408	0.875	1.095	2.732	1.707	0.718
Control Male-5	1.351	0.443	0.298	0.454	0.474	1.061	0.265
Control Male-6	3.006	1.161	0.694	1.042	2.381	1.706	0.621
LS_1 Female-1	2.169	0.809	0.435	0.685	1.512	1.731	0.449
LS_1 Female-2	2.277	0.917	0.488	0.735	1.472	1.869	0.524
LS_1 Female-3	3.227	1.196	0.751	0.917	2.081	2.255	0.573
LS_1 Male-1	4.407	1.544	0.910	1.200	2.961	2.774	0.793
LS_1 Male-2	4.639	1.470	0.875	1.185	2.781	2.633	0.918
LS_1 Male-3	3.543	1.331	0.835	0.985	2.180	2.153	0.666
LS_7 Female-1	3.874	1.068	0.618	1.560	1.466	2.879	0.711
LS_7 Female-2	4.853	1.687	1.018	1.352	2.935	2.172	0.849
LS_7 Female-3	5.054	1.754	1.093	1.379	3.221	2.335	0.949
LS_7 Female-4	1.503	0.579	0.338	0.687	1.114	1.823	0.319
LS_7 Male-1	1.868	0.525	0.324	0.775	0.745	1.899	0.423
LS_7 Male-2	3.839	1.133	0.664	1.099	1.799	2.132	0.899

Table S3: Metabolite concentrations (μmol/L of extract) in rat retina.

(cont.)

Sample ID	PC ae C38:2	PC ae C38:3	PC ae C38:4	PC ae C38:5	PC ae C38:6	PC ae C40:1	PC ae C40:2
Control Female-1	0.117	0.150	0.431	0.816	0.908	0.598	0.145
Control Female-2	0.303	0.610	1.422	2.501	2.794	1.587	0.373
Control Female-3	0.187	0.274	0.858	1.525	1.662	0.940	0.224
Control Female-4	0.267	0.441	1.075	1.879	2.271	1.626	0.376
Control Female-5	0.205	0.329	0.697	1.293	1.519	1.067	0.255
Control Female-6	0.197	0.345	0.878	1.411	1.660	1.263	0.305
Control Male-1	0.203	0.391	0.859	1.273	1.504	0.746	0.204
Control Male-2	0.419	1.287	1.187	1.704	1.855	1.209	0.339
Control Male-3	0.174	0.516	0.568	0.610	1.216	0.979	0.280
Control Male-4	0.357	0.662	1.636	2.710	3.002	1.643	0.407
Control Male-5	0.168	0.470	0.650	0.685	1.237	1.222	0.255
Control Male-6	0.346	0.726	1.260	2.222	2.698	1.756	0.434
LS_1 Female-1	0.253	0.472	0.942	1.282	1.862	1.380	0.333
LS_1 Female-2	0.327	0.637	0.966	1.353	1.719	1.579	0.319
LS_1 Female-3	0.311	0.571	1.238	1.994	2.679	1.670	0.384
LS_1 Male-1	0.432	0.660	1.790	2.961	3.522	2.607	0.637
LS_1 Male-2	0.450	0.780	1.881	2.918	3.499	2.530	0.612
LS_1 Male-3	0.328	0.510	1.305	2.369	2.910	2.042	0.506
LS_7 Female-1	0.380	0.942	1.748	2.261	3.371	2.732	0.585
LS_7 Female-2	0.382	0.677	1.722	3.085	3.790	1.872	0.485
LS_7 Female-3	0.391	0.764	1.773	3.300	3.901	2.059	0.488
LS_7 Female-4	0.177	0.314	0.690	0.992	1.510	1.640	0.370
LS_7 Male-1	0.237	0.708	0.849	1.044	1.765	1.600	0.374
LS_7 Male-2	0.393	1.049	1.332	2.040	2.482	1.482	0.335

Table S3: Metabolite concentrations (μmol/L of extract) in rat retina.

(cont.)

Sample ID	PC ae C40:3	PC ae C40:4	PC ae C40:5	PC ae C40:6	PC ae C42:0	PC ae C42:1	PC ae C42:2
Control Female-1	0.116	0.173	0.252	0.864	0.454	0.397	0.258
Control Female-2	0.480	0.582	1.164	2.819	1.269	1.145	0.635
Control Female-3	0.241	0.313	0.509	1.736	0.797	0.677	0.443
Control Female-4	0.465	0.410	0.892	2.131	1.019	1.037	0.697
Control Female-5	0.267	0.295	0.503	1.274	0.672	0.609	0.399
Control Female-6	0.307	0.347	0.646	1.702	0.765	0.741	0.539
Control Male-1	0.335	0.350	0.622	1.718	0.705	0.794	0.432
Control Male-2	1.138	0.683	2.129	2.006	1.089	1.052	0.675
Control Male-3	0.466	0.296	0.918	1.220	0.516	0.738	0.563
Control Male-4	0.647	0.648	1.181	3.222	1.188	1.480	0.754
Control Male-5	0.431	0.306	0.877	1.429	0.540	0.815	0.607
Control Male-6	0.673	0.533	1.171	2.222	1.062	1.260	0.829
LS_1 Female-1	0.436	0.367	0.883	1.819	0.953	0.804	0.492
LS_1 Female-2	0.562	0.418	1.214	1.805	0.943	0.829	0.549
LS_1 Female-3	0.531	0.525	1.119	2.616	1.271	0.997	0.635
LS_1 Male-1	0.606	0.642	1.279	3.856	1.466	2.095	1.210
LS_1 Male-2	0.679	0.748	1.573	4.183	1.516	2.063	1.277
LS_1 Male-3	0.454	0.486	1.051	3.034	1.090	1.612	0.992
LS_7 Female-1	0.710	0.658	1.884	3.277	1.204	1.267	1.057
LS_7 Female-2	0.597	0.619	1.190	3.096	1.329	1.140	0.791
LS_7 Female-3	0.728	0.664	1.547	3.369	1.389	1.271	0.859
LS_7 Female-4	0.252	0.258	0.532	1.114	0.660	0.626	0.539
LS_7 Male-1	0.519	0.416	1.337	1.673	0.659	0.891	0.837
LS_7 Male-2	0.924	0.658	1.990	2.282	0.983	0.991	0.707

Table S3: Metabolite concentrations (μmol/L of extract) in rat retina.
(cont.)

Sample ID	PC ae C42:3	PC ae C42:4	PC ae C42:5	PC ae C44:3	PC ae C44:4	PC ae C44:5	PC ae C44:6
Control Female-1	1.248	0.073	0.533	0.378	0.371	21.563	0.124
Control Female-2	3.644	0.226	2.438	1.181	0.780	71.362	0.370
Control Female-3	2.009	0.105	1.103	0.654	0.511	37.725	0.193
Control Female-4	3.206	0.304	1.757	0.944	0.799	54.957	0.312
Control Female-5	2.028	0.152	0.929	0.594	0.387	35.720	0.230
Control Female-6	2.852	0.183	1.281	0.753	0.623	41.919	0.232
Control Male-1	1.996	0.127	1.327	0.723	1.031	33.344	0.246
Control Male-2	2.940	0.203	4.067	1.253	0.849	39.566	0.470
Control Male-3	3.359	0.237	1.244	0.918	1.154	48.251	0.362
Control Male-4	4.176	0.316	2.198	1.366	1.494	56.694	0.369
Control Male-5	3.172	0.196	1.382	0.916	1.527	34.736	0.289
Control Male-6	5.139	0.269	1.885	1.419	1.657	60.318	0.397
LS_1 Female-1	2.739	0.226	1.424	0.804	0.508	49.299	0.306
LS_1 Female-2	2.224	0.179	2.320	0.721	0.549	35.555	0.245
LS_1 Female-3	3.215	0.232	2.268	0.917	0.733	51.831	0.344
LS_1 Male-1	6.345	0.275	2.371	1.741	2.371	67.876	0.418
LS_1 Male-2	6.622	0.241	2.781	1.858	3.693	61.893	0.438
LS_1 Male-3	4.209	0.187	1.932	1.201	1.755	50.442	0.340
LS_7 Female-1	6.459	0.430	2.314	1.539	2.649	64.489	0.404
LS_7 Female-2	4.009	0.308	1.849	1.167	1.340	42.865	0.300
LS_7 Female-3	5.054	0.298	2.847	1.379	1.714	54.182	0.316
LS_7 Female-4	3.204	0.191	0.706	0.637	0.674	34.634	0.191
LS_7 Male-1	4.140	0.250	1.435	1.014	3.248	37.979	0.318
LS_7 Male-2	3.731	0.247	3.081	1.083	2.715	31.811	0.335

Table S3: Metabolite concentrations (µmol/L of extract) in rat retina.

(cont.)

Sample ID	SM (OH) C14:1	SM (OH) C16:1	SM (OH) C22:1	SM (OH) C22:2	SM (OH) C24:1	SM C16:0	SM C16:1
Control Female-1	0.073	0.259	0.131	0.065	0.045	15.321	0.803
Control Female-2	0.207	0.839	0.470	0.197	0.184	40.126	1.435
Control Female-3	0.105	0.509	0.232	0.124	0.080	21.995	0.940
Control Female-4	0.162	0.598	0.287	0.157	0.106	29.161	1.271
Control Female-5	0.122	0.435	0.200	0.099	0.078	22.788	1.375
Control Female-6	0.148	0.560	0.269	0.156	0.090	25.555	1.121
Control Male-1	0.156	0.486	0.241	0.120	0.088	23.216	1.048
Control Male-2	0.180	0.746	0.338	0.012	0.146	28.576	1.374
Control Male-3	0.155	0.397	0.044	0.008	0.039	25.686	1.523
Control Male-4	0.197	0.889	0.425	0.205	0.144	41.258	1.501
Control Male-5	0.125	0.373	0.042	0.011	0.052	22.831	1.472
Control Male-6	0.212	0.656	0.208	0.102	0.059	35.616	1.994
LS_1 Female-1	0.162	0.467	0.267	0.153	0.105	24.211	1.238
LS_1 Female-2	0.131	0.484	0.363	0.101	0.129	23.202	1.028
LS_1 Female-3	0.171	0.640	0.460	0.198	0.145	31.522	1.271
LS_1 Male-1	0.222	0.925	0.493	0.242	0.199	41.316	1.515
LS_1 Male-2	0.205	0.962	0.383	0.133	0.169	40.350	1.516
LS_1 Male-3	0.159	0.679	0.397	0.211	0.144	32.171	1.318
LS_7 Female-1	0.308	1.039	0.110	0.050	0.072	47.110	2.073
LS_7 Female-2	0.266	1.005	0.434	0.239	0.133	44.599	1.583
LS_7 Female-3	0.246	1.005	0.403	0.174	0.142	44.331	1.606
LS_7 Female-4	0.143	0.359	0.068	0.022	0.049	25.479	1.602
LS_7 Male-1	0.203	0.565	0.061	0.019	0.029	31.995	1.728
LS_7 Male-2	0.204	0.717	0.273	0.074	0.127	35.558	1.724

Table S3: Metabolite concentrations (µmol/L of extract) in rat retina.

(cont.)

Sample ID	SM C18:0	SM C18:1	SM C20:2	SM C22:3	SM C24:0	SM C24:1	SM C26:0	SM C26:1
Control Female-1	18.595	1.309	0.022	0.107	1.179	1.942	0.020	0.048
Control Female-2	58.411	4.228	0.042	0.335	3.682	3.936	0.085	0.113
Control Female-3	34.184	2.240	0.035	0.173	1.893	2.356	0.045	0.071
Control Female-4	44.489	3.075	0.046	0.201	2.309	2.589	0.063	0.094
Control Female-5	32.581	2.662	0.052	0.166	1.764	2.084	0.051	0.103
Control Female-6	33.737	2.312	0.049	0.174	2.526	2.674	0.065	0.077
Control Male-1	27.954	1.854	0.033	0.155	2.073	2.772	0.031	0.078
Control Male-2	36.819	2.706	0.041	0.216	3.077	2.913	0.109	0.165
Control Male-3	19.679	2.020	0.025	0.158	0.407	0.487	0.026	0.035
Control Male-4	55.342	3.542	0.076	0.373	3.742	3.763	0.083	0.112
Control Male-5	19.737	1.582	0.026	0.156	0.458	0.580	0.035	0.029
Control Male-6	40.774	3.611	0.060	0.296	2.252	2.312	0.033	0.103
LS_1 Female-1	30.675	2.662	0.024	0.144	2.224	2.333	0.047	0.088
LS_1 Female-2	33.621	2.406	0.037	0.191	2.793	3.072	0.081	0.107
LS_1 Female-3	46.473	3.165	0.060	0.202	3.676	3.999	0.047	0.097
LS_1 Male-1	62.663	3.964	0.082	0.342	4.230	4.771	0.098	0.123
LS_1 Male-2	67.592	4.024	0.082	0.435	4.103	4.411	0.075	0.139
LS_1 Male-3	46.658	2.963	0.081	0.294	3.230	3.478	0.078	0.116
LS_7 Female-1	50.042	5.632	0.062	0.367	0.992	0.933	0.040	0.049
LS_7 Female-2	67.707	3.963	0.067	0.231	3.882	4.367	0.088	0.124
LS_7 Female-3	75.165	4.581	0.083	0.462	3.684	3.842	0.104	0.111
LS_7 Female-4	25.632	3.059	0.027	0.143	0.735	0.689	0.032	0.035
LS_7 Male-1	26.256	3.096	0.038	0.142	0.564	0.455	0.024	0.018
LS_7 Male-2	52.629	3.506	0.062	0.257	2.715	2.798	0.045	0.077

Table S3: Metabolite concentrations (μmol/L of extract) in rat retina.

(cont.)

Sample ID	H1
Control Female-1	1548.250
Control Female-2	3086.870
Control Female-3	1907.360
Control Female-4	1587.950
Control Female-5	1305.770
Control Female-6	1616.290
Control Male-1	1974.570
Control Male-2	3624.150
Control Male-3	552.045
Control Male-4	1604.060
Control Male-5	402.179
Control Male-6	1768.870
LS_1 Female-1	2529.590
LS_1 Female-2	2536.090
LS_1 Female-3	3810.070
LS_1 Male-1	2363.870
LS_1 Male-2	3112.880
LS_1 Male-3	1733.180
LS_7 Female-1	688.856
LS_7 Female-2	2763.720
LS_7 Female-3	2760.320
LS_7 Female-4	944.412
LS_7 Male-1	683.868
LS_7 Male-2	1732.930

Supplementary Table S4. Metabolic ratios in male and female control retinas. Median values were calculated for each group. P-values were calculated using the Wilcoxon rank sum test. *Legend*: ADMA= asymmetric dimethylarginine; NO= nitric oxide; Methionine-SO= methionine sulfoxide; C0= carnitine; C2=acetyl-L-carnitine; C3= propionyl-L-carnitine; lyso-PC= lysophosphatidylcholine; PC= phosphatidylcholine; SM= sphingomyelin; MUFA= mono-unsaturated fatty acid; PUFA= poly-unsaturated fatty acid; SFA= saturated fatty acid; CPT-1= carnitine palmitoyltransferase I. Asterisks (*) stand for significant ratios using a constant non-adjusted P-value threshold of 0.05. Black circles (•) stand for significant ratios using adjusted P-values to keep a false discovery rate less than 10% upon multiple comparisons.

Ratio		Metabolic significance	Sex		P-value
			Female	Male	
**Amino acid and biogenic amine metabolism**					
ADMA/Arginine		Inhibition of NO synthase	$4.71\cdot10^{-3}$	$3.31\cdot10^{-3}$	0.078
Citrulline/Arginine		Activity of NO synthase	0.15	0.11	0.055
Tyrosine/Phenylalanine		Activity of phenylalanine hydroxylase	1.46	1.99	0.025*•
Methionine-SO/Methionine		Measure of systemic oxidative stress	0.21	0.26	0.75
Spermidine/Putrescine		Activity of spermidine synthase	3.62	3.77	0.52
**Lipid metabolism**					
(C2 + C3)/C0		Measure of the overall ß-oxidation activity	0.32	0.26	0.015*•
C2/C0		Measure of ß-oxidation of even numbered fatty acids	0.30	0.25	0.026*•
(C16 + C18)/C0		Activity of CPT-I, rate limiting step in the mitochondrial uptake of FA	$7.29\cdot10^{-3}$	$6.05\cdot10^{-3}$	0.31
Total dicarboxy-acylcarnitines/ total acylcarnitines		Indicator of ω-oxidation of fatty acids	$9.47\cdot10^{-2}$	$8.19\cdot10^{-2}$	0.025*•
Total lyso PC/Total PC		Indicator of phospholipase activity	$4.16\cdot10^{-2}$	$4.24\cdot10^{-2}$	0.75
Lysophosphatidylcholine	MUFA/SFA	Measure of the activity of fatty acid desaturases	0.33	0.30	0.15
	PUFA/MUFA		0.39	0.40	0.42
	PUFA/SFA		0.13	0.12	0.26
Phosphatidylcholine	MUFA/SFA		1.70	1.64	0.42
	PUFA/MUFA		1.49	1.49	0.87
	PUFA/SFA		2.53	2.59	1.0
Sphingomyelin	MUFA/SFA		0.11	0.11	0.42
	PUFA/MUFA		$4.98\cdot10^{-2}$	$4.55\cdot10^{-2}$	0.42
	PUFA/SFA		$5.58\cdot10^{-3}$	$5.12\cdot10^{-3}$	0.75

Supplementary Table S5. Metabolic ratios between in control and light-treated retinas. Median values were calculated for each group. P-values were calculated using non-parametric Kruskal-Walis test for multiple group comparison. Legend: C0= carnitine; C2= acetyl-L-carnitine; C3= propionyl-L-carnitine; C16= hexadecanoyl-L-carnitine; C18= Octadecanoyl-L-carnitine; ADMA= asymmetric dimethylarginine; NO= nitric oxide; Methionine-SO= methionine sulfoxide; lyso PC= lysophosphatidylcholine; PC= phosphatidylcholine; SM sphingomyelin MUFA= mono-unsaturated fatty acid; PUFA= poly-unsaturated fatty acid; SFA= saturated fatty acid; CPT-1= carnitine palmitoyltransferase I. Asterisks (*) stand for significant ratios using a constant non-adjusted P-value threshold of 0.05. Black circles (•) stand for significant ratios using adjusted P-values to keep a false discovery rate less than 10% upon multiple comparisons.

Ratio		Metabolic significance	Group			P-value
			Controls	LS_1	LS_7	
**Amino acid and biogenic amine metabolism**						
ADMA/Arginine		Inhibition of NO synthase	$4.09\cdot10^{-3}$	$5.62\cdot10^{-3}$	$5.39\cdot10^{-3}$	0.16
Citrulline/Arginine		Activity of NO synthase	0.14	0.12	0.23	0.025*•
Tyrosine/Phenylalanine		Activity of phenylalanine hydroxylase	1.76	1.63	1.51	0.21
Methionine-SO/Methionine		Measure of systemic oxidative stress	0.21	0.32	0.52	0.52
Spermidine/Putrescine		Activity of spermidine synthase	3.74	1.73	3.41	0.11
**Lipid metabolism**						
(C2 + C3)/C0		Measure of the overall ß-oxidation activity	0.29	0.28	0.31	0.86
C2/C0		Measure of ß-oxidation of even numbered fatty acids	0.27	0.27	0.29	0.85
(C16 + C18)/C0		Activity of CPT-I, rate limiting step in the mitochondrial uptake of FA	$6.73\cdot10^{-3}$	$13.64\cdot10^{-3}$	$6.67\cdot10^{-3}$	0.18
Total dicarboxy-acylcarnitines/ total acylcarnitines		Indicator of ω-oxidation of fatty acids	$8.81\cdot10^{-2}$	$7.90\cdot10^{-2}$	$9.6\cdot10^{-2}$	0.058
Total lyso PC/Total PC		Indicator of phospholipase activity	$4.16\cdot10^{-2}$	$4.00\cdot10^{-2}$	$3.97\cdot10^{-2}$	0.91
Lyso PC	MUFA/SFA	Measure of the activity of fatty acid desaturases	0.31	0.29	0.31	0.33
	PUFA/MUFA		0.39	0.47	0.43	0.012*•
	PUFA/SFA		0.13	0.14	0.13	0.23
PC	MUFA/SFA		1.69	1.69	1.86	0.011*•
	PUFA/MUFA		1.49	1.59	1.44	0.43
	PUFA/SFA		2.53	2.67	2.77	0.44
SM	MUFA/SFA		0.114	0.117	0.104	0.029*•
	PUFA/MUFA		$4.8\cdot10^{-2}$	$5.0\cdot10^{-2}$	$4.3\cdot10^{-2}$	0.32
	PUFA/SFA		$5.6*10^{-3}$	$5.7*10^{-3}$	$4.5*10^{-3}$	0.048

Supplementary Table S6. Metabolites excluded from the statistical analysis.

The present table of 23 metabolites represents the list of compounds that were not considered for the statistical analysis because more than 20% of their concentration values were below the lower limit of quantitation or above the upper limit of quantification is given here after. Concentration of all of the metabolites listed here, except for amino acids (*), were below the lower limit of quantification (LLOQ).

Acylcarnitines	Amino acids*	Biogenic amines	Lyso PC	PC
C10	Asp	alpha-AAA	lysoPC a C14:0	PC aa C26:0
C10:1	Gln	DOPA		PC aa C40:1
C10:2	Lys	Nitro-Tyr		
C12-DC				
C14:1-OH				
C16:1-OH				
C16:2-OH				
C18:1-OH				
C3-OH				
C5-M-DC				
C5:1-DC				
C6:1				
C7-DC				
C9				
